# Agesasines A and B, Bromopyrrole Alkaloids from Marine Sponges *Agelas* spp

**DOI:** 10.3390/md18090455

**Published:** 2020-08-30

**Authors:** Sanghoon Lee, Naonobu Tanaka, Sakura Takahashi, Daisuke Tsuji, Sang-Yong Kim, Mareshige Kojoma, Kohji Itoh, Jun’ichi Kobayashi, Yoshiki Kashiwada

**Affiliations:** 1 Graduate School of Pharmaceutical Sciences, Tokushima University, Tokushima 770-8505, Japan; sanghoon_lee@sfu.ca (S.L.); c402031011@tokushima-u.ac.jp (S.T.); dtsuji@tokushima-u.ac.jp (D.T.); kitoh@tokushima-u.ac.jp (K.I.); 2 Department of Chemistry, Simon Fraser University, Burnaby, BC V5A 1S6, Canada; 3 Faculty of Pharmaceutical Sciences, Health Sciences University of Hokkaido, Tobetsu 061-0293, Japan; kim@hoku-iryo-u.ac.jp (S.-Y.K.); kojoma@hoku-iryo-u.ac.jp (M.K.); 4 Graduate School of Pharmaceutical Sciences, Hokkaido University, Sapporo 060-0812, Japan; jkobay@pharm.hokudai.ac.jp

**Keywords:** agesasines, bromopyrrole alkaloid, marine sponge, *Agelas*

## Abstract

Exploration for specialized metabolites of Okinawan marine sponges *Agelas* spp. resulted in the isolation of five new bromopyrrole alkaloids, agesasines A (**1**) and B (**2**), 9-hydroxydihydrodispacamide (**3**), 9-hydroxydihydrooroidin (**4**), and 9*E*-keramadine (**5**). Their structures were elucidated on the basis of spectroscopic analyses. Agesasines A (**1**) and B (**2**) were assigned as rare bromopyrrole alkaloids lacking an aminoimidazole moiety, while **3**–**5** were elucidated to be linear bromopyrrole alkaloids with either aminoimidazolone, aminoimidazole, or *N*-methylated aminoimidazole moieties.

## 1. Introduction

A number of structurally unique bioactive specialized metabolites have been isolated from marine sources including sponges, algae, cnidarians, and marine microorganisms, etc. [1]. To date, more than 8000 species of marine sponges (phylum Porifera) have been found under the sea throughout tropical, temperate, and polar area [2]. Marine sponges utilize some of their specialized metabolites as chemical defenses against predator attacks, microbial infections, biofouling, and overgrowth of other sessile organisms [3,4]. On the other hand, natural products isolated from marine sponges are recognized as an attractive source of leads for therapeutic agents due to a diversity of their chemical structures and biological activities.

Marine sponges belonging to the genus *Agelas* are known to be a rich source of bromopyrrole alkaloids and diterpene alkaloids that have been used as a taxonomically characteristic maker [5]. In our search for structurally unique marine natural products [6,7,8], we have recently reported the isolation of diterpene alkaloids from the extracts of a marine sponge *Agelas* spp. [9]. As part of this research project, we have investigated another specimen of Agelas marine sponges, which resulted in the isolation of five new bromopyrrole alkaloids (**1**–**5**). Among others, agesasines A (**1**) and B (**2**) are rare bromopyrrole alkaloids lacking an aminoimidazole moiety, from the point of view that typical bromopyrrole alkaloids consist of a brominated pyrrolecarboxamide moiety and an aminoimidazole moiety linked through a C_3_ unit. Herein, we describe the isolation and structure elucidation of **1**–**5**.

## 2. Results and Discussion

### 2.1. Isolation of ***1**–**5*** from Marine Sponges Agelas spp.

**Figure 1:**
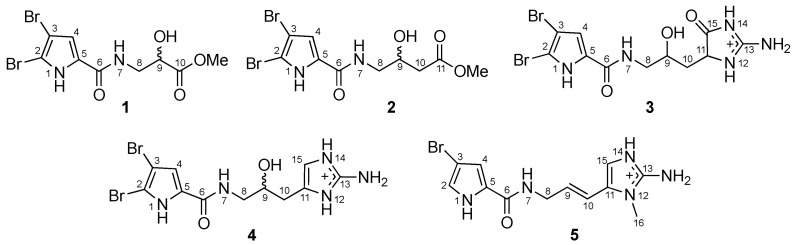
Structures of agesasines A (**1**) and B (**2**), 9-hydroxydihydrodispacamide (**3**), 9-hydroxydihydrooroidin (**4**), and 9*E*-keramadine (**5**).

Two specimens of the marine sponge *Agelas* spp. (SS-516 and SS-1302) were separately extracted with MeOH to give extracts, each of which was partitioned between *n*-hexane and 90% MeOH aq. Repeated chromatographic separations of the 90% MeOH aq.-soluble materials from SS-516 gave two new bromopyrrole alkaloids, agesasines A (**1**, 2.5 mg) and B (**2**, 2.2 mg) (Figure 1) together with two known bromopyrrole alkaloids, tauroacidin A [10] and taurodispacamide A [11]. In contrast, the 90% MeOH aq.-soluble materials of SS-1302 were further partitioned with *n*-BuOH and water. The *n*-BuOH-soluble materials were separated by column chromatographies to give three new bromopyrrole alkaloids, 9-hydroxydihydrodispacamide (**3**, 5.0 mg), 9-hydroxydihydrooroidin (**4**, 2.1 mg), and 9*E*-keramadine (**5**, 3.1 mg) (Figure 1), together with four known alkaloids, oroidin [12,13], keramadine [14], 2-bromo-9,10-dihydrokeramadine [15], and nagelamide L [16].

### 2.2. Structure Elucidation of ***1**–**5***

**Figure 2:**
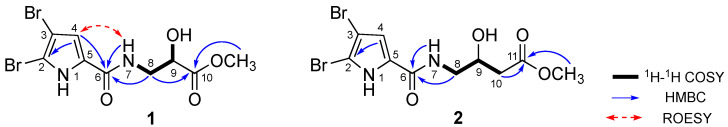
Key two-dimensional (2D) NMR correlations for agesasines A (**1**) and B (**2**).

Agesasine A (**1**) displayed ion peaks at *m*/*z* 391, 393, and 395 (1:2:1), suggesting the presence of two bromine atoms in **1**. The molecular formula of **1**, C_9_H_10_N_2_O_4_Br_2_, was determined by the high-resolution electrospray ionization mass spectrometry (HRESIMS) (*m*/*z* 390.89045 [M + Na]^+^, Δ − 0.05 mmu). The ^1^H and ^13^C NMR spectra (Table 1) displayed the signals of one sp^3^ methine, one sp^3^ methylene, one methoxy group, and one carboxy carbon as well as resonances assignable to a 2,3-dibromopyrrole carboxamide moiety (N-1~N-7). Analysis of the ^1^H-^1^H correlation spectroscopy (COSY) spectrum revealed the connectivities from 7-NH to 9-OH (Figure 2), while heteronuclear multiple bond coherence (HMBC) correlations for methoxy protons and H_2_-8 with C-10 suggested the presence of a methoxy carbonyl group at C-9. Thus, the planar structure of agesasine A (**1**) was elucidated as shown in Figure 2. Agesasine B (**2**) showed an ion peak at *m/z* 380.9088 ([M − H]^−^, Δ + 0.2 mmu), corresponding to the molecular formula of C_10_H_12_N_2_O_4_Br_2_. The 1D NMR spectra of **2** (Table 1) were closely correlated to those of **1**, except for the presence of an additional sp^3^ methylene signal (CH_2_-10) in **2**. The methylene protons (H_2_-10) showed a ^1^H-^1^H COSY cross-peak with H-9 and an HMBC correlation with a methoxy carbonyl carbon (C-11), suggesting the planar structure of **2** as shown in Figure 2.

The racemic nature of agesasines A (**1**) and B (**2**) indicated by their specific rotation values being nearly zero prompted us to perform the optical resolutions of **1** and **2**. The analysis of **1** using the reversed phase chiral high performance liquid chromatography (HPLC) gave a pair of peaks in the integral ratio of ca. 1:1, indicating agesasine A (**1**) to be a racemate. Agesasine B (**2**) was also deduced to be a racemate, although the optical resolution could not be achieved in spite of attempts being made at various separation conditions.

**Figure 3:**
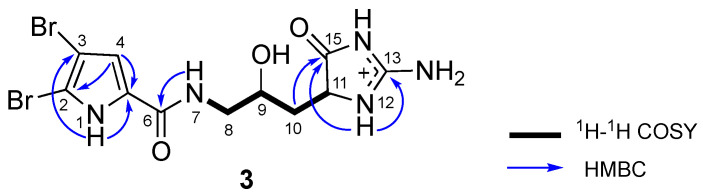
Selected 2D NMR correlations for 9-hydroxydihydrodispacamide (**3**).

9-Hydroxydihydrodispacamide (**3**) was obtained as a pale yellow amorphous solid. The HRESIMS showed an ion peak at *m*/*z* 443.92824 ([M − H + Na]^+^, Δ − 0.04 mmu), suggesting the molecular formula of C_11_H_14_N_5_O_3_Br_2_. The ^1^H and ^13^C NMR spectra of **3** (Table 2) were similar to those of a known linear bromopyrrole alkaloid, dihydrodispacamide [17], except for the presence of an oxygenated methine signal (CH-9) in **3**. Therefore, **3** was deduced to be a hydroxylated derivative of dihydrodispacamide. The presence of the hydroxy group at C-9 was confirmed by ^1^H-^1^H COSY cross-peaks of H_2_-8/H-9 and H-9/H_2_-10 (Figure 3). The relative configuration of **3** was not assigned in this study, while the racemic nature of **3** was confirmed by HPLC analysis with chiral column with a similar manner as for **1**.

**Figure 4:**
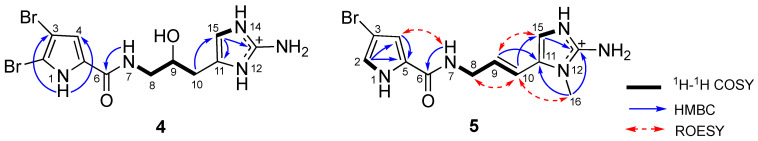
Selected 2D NMR correlations for 9-hydroxydihydrooroidin (**4**) and 9*E*-keramadine (**5**).

9-Hydroxydihydrooroidin (**4**) was obtained as a pale yellow amorphous solid. Although the ^1^H and ^13^C NMR spectra (Table 2) implied that **4** was a bromopyrrole alkaloid related to dihydrooroidin [17], the signals of an oxygenated methine (CH-9, δ_H_ 3.76, and δ_C_ 68.4) were observed in **4**. In the ^1^H-^1^H COSY spectrum, the oxygenated methine proton (H-9) showed cross-peaks with H_2_-8 and H_2_-10 (Figure 4). Based on the above findings and the molecular formula of **4**, C_11_H_14_N_5_O_2_Br_2_, obtained by the HRESIMS (*m*/*z* 405.9510 [M]^+^, Δ − 0.4 mmu), **4** was assigned as 9-hydroxydihydrooroidin (Figure 1). A nearly zero value of the specific rotation indicated **4** to be a racemate, which was supported by the fact that **4** showed no cotton effect in the electronic circular dichroism (ECD) spectrum.

9*E*-Keramadine (**5**) displayed the ^1^H and ^13^C NMR spectra (Table 2) similar to those of a known bromopyrrole alkaloid possessing a 3-bromopyrrolecarboxamide moiety, keramadine [14]. The HRESIMS revealed the molecular formula of **5** to be C_12_H_15_N_5_OBr, which was identical to that of keramadine. However, the ^3^*J* (H-9/H-10) value (*J* = 16.1 Hz) in **5** indicated the geometry of the double bond to be *E*, whereas keramadine has the *Z*-olefin. The *E*-geometry was further underpinned by rotating frame nuclear Overhauser effect spectroscopy (ROESY) correlations for H-9/H-15 and H_2_-8/H-10 (Figure 4). This is the first report of 9*E*-keramadine from a natural source, although the synthesis of 9*E*-keramadine has been reported to date [18].

Bromopyrrole alkaloids isolated from marine sponges have attracted the interest of researchers due to their diverse chemical structures. Various intriguing biological activities of bromopyrrole alkaloids leading drug discovery such as cytotoxic, antibacterial (antibiofilm), and protein kinase C modulating activities have been reported [19,20]. We have also reported the isolation of antimicrobial bromopyrrole alkaloids to date [6]. In this study, the antiproliferative activity of **1**–**5** against human cancer cell lines (HeLa, A549, and MCF7) were evaluated, showing no cytotoxicity against all cell lines (IC_50_ > 100 µM) (Figures S43–S45).

In conclusion, five new bromopyrrole alkaloids, agesasines A (**1**) and B (**2**), 9-hydroxydihydrodispacamide (**3**), 9-hydroxydihydrooroidin (**4**), and 9*E*-keramadine (**5**) were isolated from Okinawan marine sponges *Agelas* spp. Typical bromopyrrole alkaloids such as oroidin [12,13] and keramadine [14] consist of a mono or dibrominated pyrrolecarboxamide moiety and an aminoimidazole moiety linked through a C_3_ unit. In contrast, agesasines A (**1**) and B (**2**) are rare bromopyrrole alkaloids lacking an aminoimidazole moiety, whereas **1** and **2** might be artifacts during the extraction and isolation process with acidic condition. Few alkaloids with such structural feature have been isolated from marine sponges *Agelas* spp. collected off the South China Sea [21,22].

## 3. Materials and Methods

### 3.1. General Procedures

Optical rotations were obtained on a JASCO P-2200 digital polarimeter (JASCO Co., Tokyo, Japan). UV spectra were recorded on a Hitachi U-3900H spectrophotometer (Hitachi, Ltd., Tokyo, Japan). NMR spectra were measured by a Bruker AVANCE-500 instrument (Bruker, Billerica, MA, USA) using tetramethylsilane as an internal standard. HRESIMS were recorded on a Waters LCT PREMIER 2695 (Waters Co., Milford, MA, USA) and a JEOL JMS-T100LP (JEOL, Ltd., Tokyo, Japan). Column chromatography was performed with silica gel 60 N (Kanto Kagaku, Tokyo, Japan) and Diaion HP-20 (Mitsubishi Chemical, Tokyo, Japan). Medium pressure liquid chromatography (MPLC) was carried out on Toyopearl HW-40F (TOSOH Co., Tokyo, Japan), MCI gel CHP20P (Mitsubishi Chemical, Tokyo, Japan), and Biotage SNAP Cartridge KP-C18-HS (Biotage, Uppsala, Sweden).

### 3.2. Materials

The marine sponges *Agelas* spp. were collected off Kerama Islands, Okinawa, and identified by one of the authors (N.T.). The voucher specimens (SS-516 and SS-1302) were deposited in the Graduate School of Pharmaceutical Sciences, Tokushima University.

### 3.3. Extraction and Isolation

The marine sponges *Agelas* spp. SS-516 (5.22 kg, wet weight) and SS-1302 (3.42 kg, wet weight) were separately extracted with MeOH to give the extracts (197.1 and 376.3 g, respectively), each of which was partitioned with *n*-hexane and 90% MeOH aq. The 90% MeOH aq.-soluble materials of SS-516 were separated by column chromatography on Diaion HP-20 (MeOH/H_2_O, 0:100–100:0) to give six fractions (frs. 1–6). Fr. 3 was subjected to silica gel column chromatography (CHCl_3_/MeOH/TFA, 95:5:0.1–80:20:0.1) to yield 12 fractions (frs. 3.1–3.12). Fr. 3.7 was applied to ODS MPLC (MeCN/H_2_O/TFA, 5:95:0.1–80:20:0.1), and then purified by ODS HPLC (YMC Hydrosphere C18, ϕ20 × 250 mm, MeCN/H_2_O/TFA, 35:65:0.1) to furnish agesasines A (**1**, 2.5 mg) and B (**2**, 2.2 mg). Separation of fr. 3.11 by ODS MPLC (MeCN/H_2_O/TFA, 5:95:0.1–80:20:0.1) gave five fractions (frs. 3.11.1–3.11.5). Tauroacidin A (124.1 mg) and taurodispacamide A (34.5 mg) were purified from fr. 3.11.3 by ODS MPLC (MeCN/H_2_O/TFA, 20:80:0.1).

The 90% MeOH aq.-soluble materials of SS-1302 were further partitioned between *n*-BuOH and water. The *n*-BuOH-soluble materials (58.0 g) were applied to silica gel column chromatography (CHCl_3_/MeOH/TFA, 9:1:0.1–5:5:0.1) to give six fractions (frs. 1′–6′) including oroidin (17.1 g, fr. 3′). Fr. 4′ was subjected to MPLC on a Toyopearl HW-40F column (MeOH/H_2_O/TFA, 10:90:0.1–90:10:0.1), an MCI gel CHP 20P column (MeOH/H_2_O/TFA, 10:90:0.1–90:10:0.1) to yield seven fractions (frs. 4′.4.1–4′.4.7). Fr. 4′.4.3 was loaded to MPLC on an ODS column (MeCN/H_2_O/TFA, 10:90:0.1–60:40:0.1) to give six fractions (frs. 4′.4.3.1–4′.4.3.6), and then fr. 4′.4.3.3 was purified by ODS HPLC (COSMOSIL 5C_18_-MS-Ⅱ, ϕ 20 × 250 mm, MeCN/H_2_O/TFA, 17:83:0.1). Further purification of fr. 4′.4.3.3.2 on ODS HPLC (YMC Hydrosphere C18, ϕ 10 × 250 mm, MeCN/H_2_O/TFA, 13:87:0.1) afforded 9-hydroxydihydrodispacamide (**3**, 5.0 mg), 9*E*-keramadine (**5**, 3.1 mg), and keramadine (6.7 mg). 9-Hydroxydihydrooroidin (**4**, 2.1 mg) was isolated from fr. 4′4.3.3.3 by ODS HPLC (YMC Hydrosphere C18, ϕ 10 × 250 mm, MeCN/H_2_O/TFA, 13:87:0.1). Fr. 4′.4.4 was subjected to ODS MPLC (MeCN/H_2_O/TFA, 10:90:0.1–50:50:0.1) and then ODS HPLC (YMC Hydrosphere C18, ϕ 10 × 250 mm, MeCN/H_2_O/TFA, 15:85:0.1) to furnish 2-bromo-9,10-dihydrokeramadine (2.1 mg). Fr. 5′ was applied to MPLC on a Toyopearl HW-40F column (MeOH/H_2_O/TFA, 10:90:0.1–90:10:0.1) to give eight fractions (frs. 5′.1–5′.8). Fr. 5′ was passed through an MCI gel CHP 20P column (MeOH/H_2_O/TFA, 10:90:0.1–100:0:0.1) and an ODS column (MeOH/H_2_O/TFA, 10:90:0.1–0:10:0.1) to afford nagelamide L (187.5 mg). Tauroacidin A and nagelamide L did not show optical rotations.

*Agesasine A* (**1**): Pale yellow amorphous solid; [α]${}_{D}^{28}$ 0 (*c* 0.10, MeOH); UV (MeOH) λ_max_ 275 (ε 4900) nm; ^1^H and ^13^C NMR data (Table 1); ESIMS: *m*/*z* 391, 393, and 395 (1:2:1), [M + Na]^+^; HRESIMS: *m/z* 390.89045 [M + Na]^+^ (calcd for C_9_H_10_N_2_O_4_Na^79^Br_2_, 390.89050).

*Agesasine B* (**2**): Pale yellow amorphous solid; [α]${}_{D}^{28}$ 0 (*c* 0.10, MeOH); UV (MeOH) λ_max_ 274 (ε 3100) nm; ^1^H and ^13^C NMR data (Table 1); ESIMS: *m*/*z* 381, 383, and 385 (1:2:1), [M − H]^−^; HRESIMS: *m/z* 380.9088 [M − H]^−^ (calcd for C_10_H_11_N_2_O_4_^79^Br_2_, 380.9086).

*9-Hydroxydihydrodispacamide* (**3**): Pale yellow amorphous solid; [α]${}_{D}^{27}$ 0 (*c* 0.10, MeOH); UV (MeOH) λ_max_ 223 (ε 3900) and 275 (3400) nm; ^1^H and ^13^C NMR data (Table 2); ESIMS: *m*/*z* 444, 446, and 448 (1:2:1), [M − H + Na]^+^; HRESIMS: *m*/*z* 443.92824 [M − H + Na]^+^ (calcd for C_11_H_13_N_5_O_3_Na^79^Br_2_, 443.92828).

*9-Hydroxydihydrooroidin* (**4**): Pale yellow amorphous solid; [α]${}_{D}^{27}$ 0 (*c* 0.10, MeOH); UV (MeOH) λ_max_ 276 (ε 3900) nm; ^1^H and ^13^C NMR data (Table 2); ESIMS: *m*/*z* 406, 408, and 410 (1:2:1), [M]^+^; HRESIMS: *m/z* 405.9510 [M]^+^ (calcd for C_11_H_14_N_5_O_2_^79^Br_2_, 405.9514).

*9*E*-Keramadine* (**5**): Pale yellow amorphous solid; UV (MeOH) λ_max_ 271 (ε 3300) nm; ^1^H and ^13^C NMR data (Table 2); ESIMS: *m*/*z* 324 and 326 (1:1), [M]^+^; HRESIMS: *m*/*z* 324.04592 [M]^+^ (calcd for C_12_H_15_N_5_O^79^Br, 324.04600).

### 3.4. Optical Resolutions of ***1**–**3***

Optical resolutions of agesasine A (**1**) and 9-hydroxydihydrodispacamide (**3**), were performed on chiral HPLC (Chiral ART Cellulose-SB, YMC, ϕ 4.6 × 250 mm, flow rate 0.5 mL/min, UV detection 254 nm) at 35 °C with elution of MeOH/MeCN/H_2_O/H_3_PO_4_ (30:10:60:0.1 for **1**; 8:2:90:0.1 for **3**) to give enantiomers in the integral ratio of ca. 1:1 (*t_R_* 27.5 and 29.0 min for **1**; *t_R_* 12.5 and 14.3 min for **3**) in each case. The separations of enantiomers were confirmed by MS analyses. Separated peaks for enantiomers of agesasine B (**2**) could not be obtained in any condition in this study.

### 3.5. Evaluation for Antiproliferative Activity of ***1**–**5***

New bromopyrrole alkaloids **1**–**5** were evaluated for their antiproliferative activity against human cancer cell lines (HeLa, A549, and MCF7) according to the following procedure. The human cancer cell lines were cultured in Dulbecco’s modified eagle medium (DMEM) supplemented with 5% fetal bovine serum (FBS). All cells were incubated at 37 °C in a humidified atmosphere with 5% CO_2_–95% air. Cells were seeded at 1 × 10^4^ cells/well in a 96-well plate and preincubated for 24 h. Test samples were dissolved in small amounts of DMSO and diluted in the appropriate culture medium (final concentration of DMSO < 1%). After removal of the preincubated culture medium, 100 µL of medium containing various concentrations of test compound was added and further incubated for 48 h. A cell proliferation assay was performed with the Cell Counting Kit-8 (WST-8; Dojindo, Japan) according to the manufacturer’s instruction. Briefly, the WST-8 reagent solution (10 µL) was added to each well of a 96-well microplate containing 100 µL of cells in the culture medium at various densities, and the plate was incubated for 2 h at 37 °C. Absorbance was measured at 450 nm using a microplate reader. Cisplatin was used as a positive control, whose IC_50_ values against HeLa, A549, and MCF7 cells were 11.7, 7.2, and 52.4 mM, respectively.

## Figures and Tables

**Table 1:** One-dimensional (1D) NMR data for agesasines A (**1**) and B (**2**) in DMSO-*d*_6_.

Position	1		2	
	^13^C	^1^H (*J* in Hz)	^13^C	^1^H (*J* in Hz)
1	–	12.67 (brs)	–	12.65 (brs)
2	104.8	–	104.7	–
3	98.0	–	98.0	–
4	113.1	6.93 (brs)	113.0	6.93 (d, 2,7)
5	128.1	–	128.3	–
6	159.3	–	159.3	–
7	–	8.20 (t, 5.8)	–	8.12 (t, 5.5)
8	42.7	3.46, 3.36 (each 1 H, m)	44.9	3.20 (2 H, m)
9	69.3	4.17 (q, 6.1)	66.6	3.99 (m)
10	173.1	–	40.6	2.49 (m), 2.27 (dd, 15.2, 8.8)
11	–	–	171.8	–
9-OH	–	5.71 (d, 5.9)	–	nd
OMe	51.8	3.61 (3 H, brs)	51.4	3.56 (3 H, brs)

nd: Not detected.

**Table 2:** 1D NMR data for 9-hydroxydihydrodispacamide (**3**), 9-hydroxydihydrooroidin (**4**), and 9*E*-keramadine (**5**) in DMSO-*d*_6_.

Position	3		4		5	
	^13^C	^1^H (*J* in Hz)	^13^C	^1^H (*J* in Hz)	^13^C	^1^H (*J* in Hz)
1	–	12.66 (brs)	–	12.66 (brs)	–	11.83 (brs)
2	104.7	–	104.8	–	121.6	6.98 (dd, 2.9, 1.6)
3	98.0	–	98.2	–	95.2	–
4	113.1	6.94 (d, 2.8)	113.2	6.86 (s)	111.8	6.92 (s)
5	128.3	–	128.4	–	126.9	–
6	159.3	–	159.4	–	159.7	–
7	–	8.15 (t, 5.9)	–	8.19 (t, 5.6)	–	8.40 (t, 5.5)
8	45.3	3.18 (2 H, m)	44.8	3.23 (m), 3.16 (m)	40.4	3.99 (2 H, t, 5.5)
9	66.3	3.79 (m)	68.4	3.76 (m)	130.8	6.19 (dt, 16.1, 5.5)
10	34.8	1.96 (ddd, 14.4, 5.5, 2.6) 1.71 (ddd, 14.4, 10.9, 5.5)	30.1	2.57 (dd, 15.2, 4.2) 2.40 (dd, 15.2, 7.8)	115.3	6.30 (d, 16.1)
11	56.8	4.34 (t, 5.5)	124.3	–	126.6	–
12	–	9.47 (brs)	–	11.95 (brs)		
13	158.2	–	147.1	–	146.9	–
14	–	nd	–	11.87 (brs)	–	12.35 (brs)
15	175.6	–	110.1	6.58 (brs)	109.4	7.14 (brs)
*N*-Me					29.8	3.38 (3 H, s)
13-NH_2_	–	nd	–	7.35 (2 H, brs)	–	7.71 (2 H, brs)

nd: Not detected.

## Supplementary Materials

The following are available online at https://www.mdpi.com/1660-3397/18/9/455/s1. Figure S1: ^1^H NMR spectrum of agesasine A (**1**) in DMSO-*d*_6_ (500 MHz), Figure S2: ^13^C NMR spectrum of agesasine A (**1**) in DMSO-*d*_6_ (125 MHz), Figure S3: ^1^H-^1^H COSY spectrum of agesasine A (**1**) in DMSO-*d*_6_ (500 MHz), Figure S4: HSQC spectrum of agesasine A (**1**) in DMSO-*d*_6_ (500 MHz), Figure S5: HMBC spectrum of agesasine A (**1**) in DMSO-*d*_6_ (500 MHz), Figure S6: ROESY spectrum of agesasine A (**1**) in DMSO-*d*_6_ (500 MHz), Figure S7: HRESIMS spectrum (pos.) of agesasine A (**1**), Figure S8: Chiral HPLC chart of agesasine A (**1**), Figure S9: ^1^H NMR spectrum of agesasine B (**2**) in DMSO-*d*_6_ (500 MHz), Figure S10: ^13^C NMR spectrum of agesasine B (**2**) in DMSO-*d*_6_ (125 MHz), Figure S11: ^1^H-^1^H COSY spectrum of agesasine B (**2**) in DMSO-*d*_6_ (500 MHz), Figure S12: HSQC spectrum of agesasine B (**2**) in DMSO-*d*_6_ (500 MHz), Figure S13: HMBC spectrum of agesasine B (**2**) in DMSO-*d*_6_ (500 MHz), Figure S14: HRESIMS spectrum (neg.) of agesasine B (**2**), Figure S15: ^1^H NMR spectrum of 9-hydroxydihydrodispacamide (**3**) in DMSO-*d*_6_ (500 MHz), Figure S16: ^13^C NMR spectrum of 9-hydroxydihydrodispacamide (**3**) in DMSO-*d*_6_ (125 MHz), Figure S17: ^1^H-^1^H COSY spectrum of 9-hydroxydihydrodispacamide (**3**) in DMSO-*d*_6_ (500 MHz), Figure S18: HSQC spectrum of 9-hydroxydihydrodispacamide (**3**) in DMSO-*d*_6_ (500 MHz), Figure S19: HMBC spectrum of 9-hydroxydihydrodispacamide (**3**) in DMSO-*d*_6_ (500 MHz), Figure S20: HRESIMS spectrum (pos.) of 9-hydroxydihydrodispacamide (**3**), Figure S21: Chiral HPLC chart of 9-hydroxydihydrodispacamide (**3**), Figure S22: ^1^H NMR spectrum of 9-hydroxydihydrooroidin (**4**) in DMSO-*d*_6_ (500 MHz), Figure S23: ^13^C NMR spectrum of 9-hydroxydihydrooroidin (**4**) in DMSO-*d*_6_ (125 MHz), Figure S24: ^1^H-^1^H COSY spectrum of 9-hydroxydihydrooroidin (**4**) in DMSO-*d*_6_ (500 MHz), Figure S25: HSQC spectrum of 9-hydroxydihydrooroidin (**4**) in DMSO-*d*_6_ (500 MHz), Figure S26: HMBC spectrum of 9-hydroxydihydrooroidin (**4**) in DMSO-*d*_6_ (500 MHz), Figure S27: HRESIMS spectrum (pos.) of 9-hydroxydihydrooroidin (**4**), Figure S28: ECD spectrum of 9-hydroxydihydrooroidin (**4**) in MeOH, Figure S29: ^1^H NMR spectrum of 9*E*-keramadine (**5**) in DMSO-*d*_6_ (500 MHz), Figure S30: ^13^C NMR spectrum of 9*E*-keramadine (**5**) in DMSO-*d*_6_ (125 MHz), Figure S31: ^1^H-^1^H COSY spectrum of 9*E*-keramadine (**5**) in DMSO-*d*_6_ (500 MHz), Figure S32: HSQC spectrum of 9*E*-keramadine (**5**) in DMSO-*d*_6_ (500 MHz), Figure S33: HMBC spectrum of 9*E*-keramadine (**5**) in DMSO-*d*_6_ (500 MHz), Figure S34: ROESY spectrum of 9*E*-keramadine (**5**) in DMSO-*d*_6_ (500 MHz), Figure S35: HRESIMS spectrum (pos.) of 9*E*-keramadine (**5**), Figure S36: ^1^H NMR spectrum of tauroacidin A in DMSO-*d*_6_ (500 MHz), Figure S37: ^1^H NMR spectrum of taurodispacamide A in DMSO-*d*_6_ (500 MHz), Figure S38: ^1^H NMR spectrum of oroidin in DMSO-*d*_6_ (500 MHz), Figure S39: ^1^H NMR spectrum of keramadine in DMSO-*d*_6_ (500 MHz), Figure S40: ^1^H NMR spectrum of 2-bromo-9,10-dihydrokeramadine in DMSO-*d*_6_ (500 MHz), Figure S41: ^1^H NMR spectrum of nagelamide L in DMSO-*d*_6_ (500 MHz), Figure S42: Structures of known bromopyrrole alkaloids, tauroacidin A, taurodispacamide A, oroidin, keramadine, 2-bromokeramadine, and nagelamide L, Figure S43: Antiproliferative activity of **1**–**5** against HeLa cells, Figure S44: Antiproliferative activity of **1**–**5** against A549 cells, Figure S45: Antiproliferative activity of **1**–**5** against MCF7 cells, Table S1: 1D and 2D NMR data for agesasine A (**1**) in DMSO-*d*_6_, Table S2: 1D and 2D NMR data for agesasine B (**2**) in DMSO-*d*_6_, Table S3: 1D and 2D NMR data for 9-hydroxydihydrodispacamide (**3**) in DMSO-*d*_6_, Table S4: 1D and 2D NMR data for 9-hydroxydihydrooroidin (**4**) in DMSO-*d*_6_, Table S5: 1D and 2D NMR data for 9*E*-keramadine (**5**) in DMSO-*d*_6_, Table S6: ^1^H NMR data for tauroacidin A and taurodispacamide A in DMSO-*d*_6_, Table S7: ^1^H NMR data for oroidin, keramadine, and 2-bromo-9,10-dihydrokeramadine in DMSO-*d*_6_, Table S8: ^1^H NMR data for nagelamide L in DMSO-*d*_6_.
