# Supplementary material for: Agesasines A and B, Bromopyrrole Alkaloids from Marine Sponges Agelas spp
Source: Mar Drugs. 2020 Aug 30;18(9):455. doi: 10.3390/md18090455 (PMC7551770; doi:10.3390/md18090455)
Supplement: Supplementary file 1 [file marinedrugs-18-00455-s001.pdf]

Agesasines A and B, Bromopyrrole Alkaloids from Marine Sponges *Agelas* spp.

Sanghoon Lee [1,2], Naonobu Tanaka [1,], Sakura Takahashi [1], Daisuke Tsuji [1], Sang-Yong Kim [3], Mareshige Kojoma [3], Kohji Itoh [1], Jun'ichi Kobayashi [4] and Yoshiki Kashiwada [1,*]*

[1] Graduate School of Pharmaceutical Sciences, Tokushima University, Tokushima 770-8505, Japan

[2] Department of Chemistry, Simon Fraser University, British Columbia V5A 1S6, Canada

[3] Faculty of Pharmaceutical Sciences, Health Sciences University of Hokkaido, Tobetsu 061-0293, Japan

[4] Graduate School of Pharmaceutical Sciences, Hokkaido University, Sapporo 060-0812, Japan

Supporting Information

Figure S1. ^{1}H NMR spectrum of agesasine A (**1**) in DMSO-d_6 (500 MHz).

Figure S2. ^{13}C NMR spectrum of agesasine A (**1**) in DMSO-d_6 (125 MHz).

Figure S3. ^{1}H-^{1}H COSY spectrum of agesasine A (**1**) in DMSO-d_6 (500 MHz).

Figure S4. HSQC spectrum of agesasine A (**1**) in DMSO-d_6 (500 MHz).

Figure S5. HMBC spectrum of agesasine A (**1**) in DMSO-d_6 (500 MHz).

Figure S6. ROESY spectrum of agesasine A (**1**) in DMSO-d_6 (500 MHz).

Figure S7. HRESIMS spectrum (pos.) of agesasine A (**1**).

Figure S8. Chiral HPLC chart of agesasine A (**1**).

Figure S9. ^{1}H NMR spectrum of agesasine B (**2**) in DMSO-d_6 (500 MHz).

Figure S10. ^{13}C NMR spectrum of agesasine B (**2**) in DMSO-d_6 (125 MHz).

Figure S11. ^{1}H-^{1}H COSY spectrum of agesasine B (**2**) in DMSO-d_6 (500 MHz).
Figure S12. HSQC spectrum of agesasine B (**2**) in DMSO-d_6 (500 MHz).
Figure S13. HMBC spectrum of agesasine B (**2**) in DMSO-d_6 (500 MHz).
Figure S14. HRESIMS spectrum (neg.) of agesasine B (**2**).
Figure S15. ^{1}H NMR spectrum of 9-hydroxydihydrodispacamide (**3**) in DMSO-d_6 (500 MHz).
Figure S16. ^{13}C NMR spectrum of 9-hydroxydihydrodispacamide (**3**) in DMSO-d_6 (125 MHz).
Figure S17. ^{1}H-^{1}H COSY spectrum of 9-hydroxydihydrodispacamide (**3**) in DMSO-d_6 (500 MHz).
Figure S18. HSQC spectrum of 9-hydroxydihydrodispacamide (**3**) in DMSO-d_6 (500 MHz).
Figure S19. HMBC spectrum of 9-hydroxydihydrodispacamide (**3**) in DMSO-d_6 (500 MHz).
Figure S20. HRESIMS spectrum (pos.) of 9-hydroxydihydrodispacamide (**3**).
Figure S21. Chiral HPLC chart of 9-hydroxydihydrodispacamide (**3**).
Figure S22. ^{1}H NMR spectrum of 9-hydroxydihydrooroidin (**4**) in DMSO-d_6 (500 MHz).
Figure S23. ^{13}C NMR spectrum of 9-hydroxydihydrooroidin (**4**) in DMSO-d_6 (125 MHz).
Figure S24. ^{1}H-^{1}H COSY spectrum of 9-hydroxydihydrooroidin (**4**) in DMSO-d_6 (500 MHz).
Figure S25. HSQC spectrum of 9-hydroxydihydrooroidin (**4**) in DMSO-d_6 (500 MHz).
Figure S26. HMBC spectrum of 9-hydroxydihydrooroidin (**4**) in DMSO-d_6 (500 MHz).
Figure S27. HRESIMS spectrum (pos.) of 9-hydroxydihydrooroidin (**4**).
Figure S28. ECD spectrum of 9-hydroxydihydrooroidin (**4**) in MeOH.
Figure S29. ^{1}H NMR spectrum of 9*E*-keramadine (**5**) in DMSO-d_6 (500 MHz).
Figure S30. ^{13}C NMR spectrum of 9*E*-keramadine (**5**) in DMSO-d_6 (125 MHz).
Figure S31. ^{1}H-^{1}H COSY spectrum of 9*E*-keramadine (**5**) in DMSO-d_6 (500 MHz).
Figure S32. HSQC spectrum of 9*E*-keramadine (**5**) in DMSO-d_6 (500 MHz).
Figure S33. HMBC spectrum of 9*E*-keramadine (**5**) in DMSO-d_6 (500 MHz).
Figure S34. ROESY spectrum of 9*E*-keramadine (**5**) in DMSO-d_6 (500 MHz).
Figure S35. HRESIMS spectrum (pos.) of 9*E*-keramadine (**5**).
Figure S36. ^{1}H NMR spectrum of tauroacidin A in DMSO-d_6 (500 MHz).
Figure S37. ^{1}H NMR spectrum of taurodispacamide A in DMSO-d_6 (500 MHz).
Figure S38. ^{1}H NMR spectrum of oroidin in DMSO-d_6 (500 MHz).
Figure S39. ^{1}H NMR spectrum of keramadine in DMSO-d_6 (500 MHz).
Figure S40. ^{1}H NMR spectrum of 2-bromo-9,10-dihydrokeramadine in DMSO-d_6 (500 MHz).
Figure S41. ^{1}H NMR spectrum of nagelamide L in DMSO-d_6 (500 MHz).
Figure S42. Structures of known bromopyrrole alkaloids, tauroacidin A, taurodispacamide A, oroidin, keramadine, 2-bromokeramadine, and nagelamide L.
Figure S43. Antiproliferative activity of **1**–**5** against HeLa cells.
Figure S44. Antiproliferative activity of **1**–**5** against A549 cells.

Figure S45. Antiproliferative activity of **1**–**5** against MCF7 cells.
Table S1. 1D and 2D NMR data for agesasine A (**1**) in DMSO-d_6.
Table S2. 1D and 2D NMR data for agesasine B (**2**) in DMSO-d_6.
Table S3. 1D and 2D NMR data for 9-hydroxydihydrodispacamide (**3**) in DMSO-d_6.
Table S4. 1D and 2D NMR data for 9-hydroxydihydrooroidin (**4**) in DMSO-d_6.
Table S5. 1D and 2D NMR data for 9-(*E*)-keramadine (**5**) in DMSO-d_6.
Table S6. ^{1}H NMR data for tauroacidin A and taurodispacamide A in DMSO-d_6.
Table S7. ^{1}H NMR data for oroidin, keramadine, and 2-bromo-9,10-dihydrokeramadine in DMSO-d_6.
Table S8. ^{1}H NMR data for nagelamide L in DMSO-d_6.

Figure S1. ^{1}H NMR spectrum of agesasine A (**1**) in DMSO-d_6 (500 MHz).

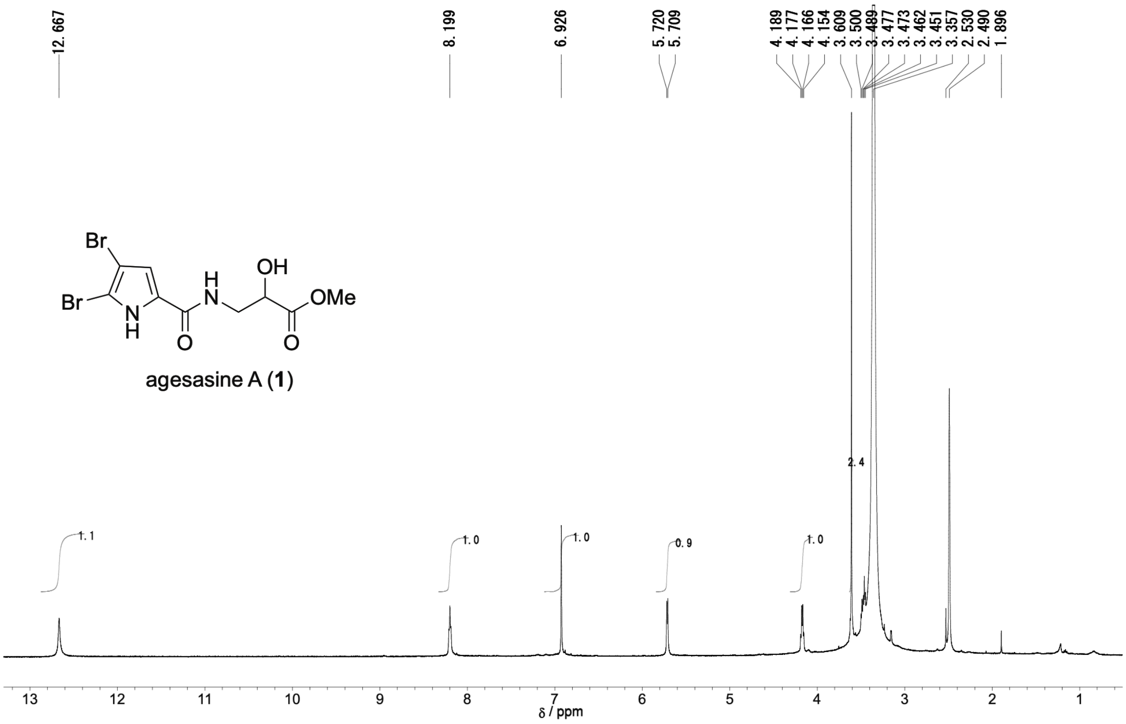

Figure S2. ^{13}C NMR spectrum of agesasine A (**1**) in DMSO-d_6 (125 MHz).

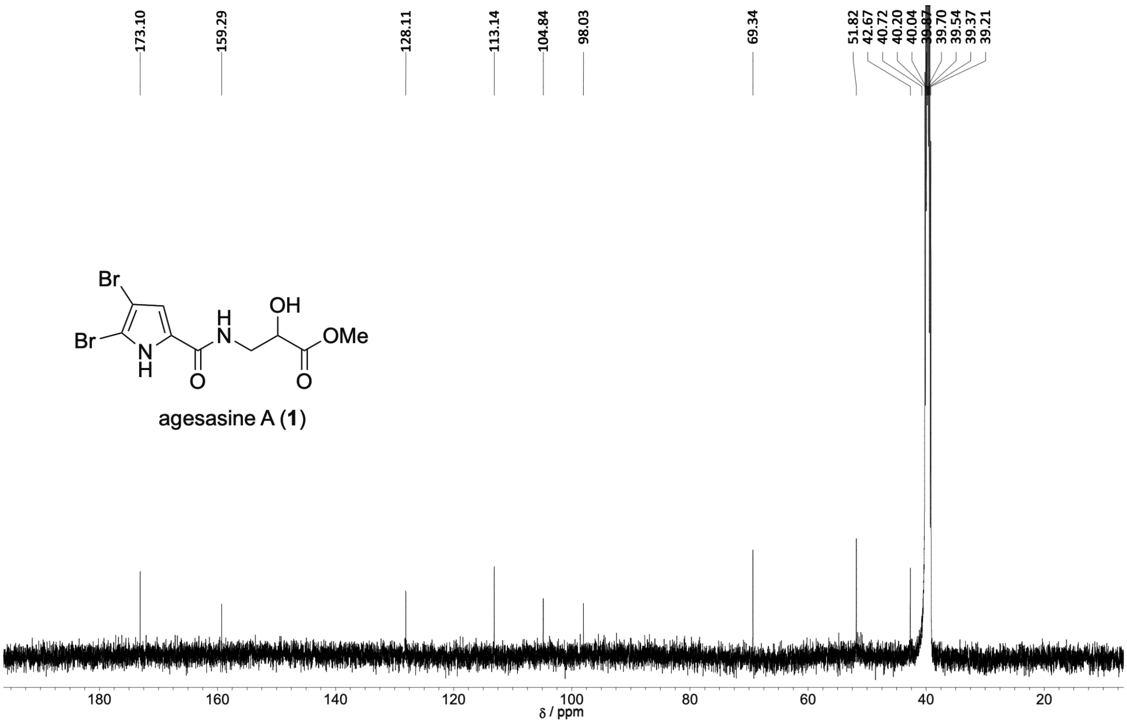

Figure S3. ^{1}H-^{1}H COSY spectrum of agesasine A (**1**) in DMSO-d_6 (500 MHz).

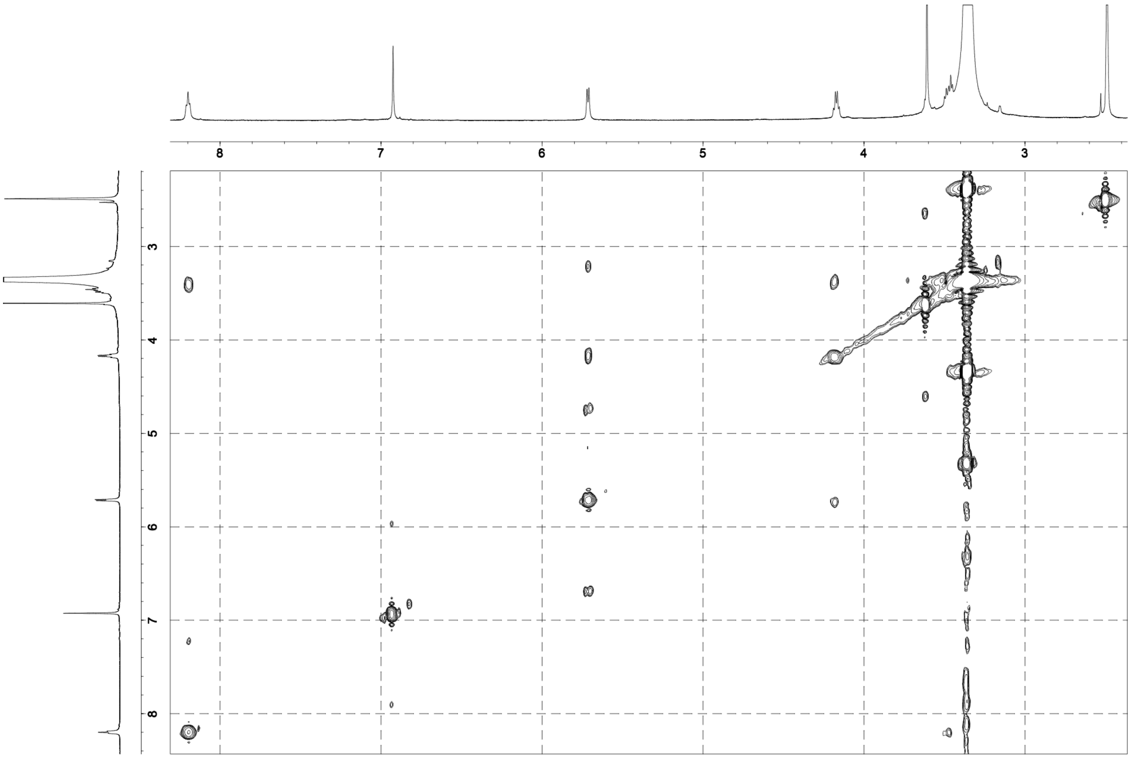

Figure S4. HSQC spectrum of agesasine A (**1**) in DMSO-d_6 (500 MHz).

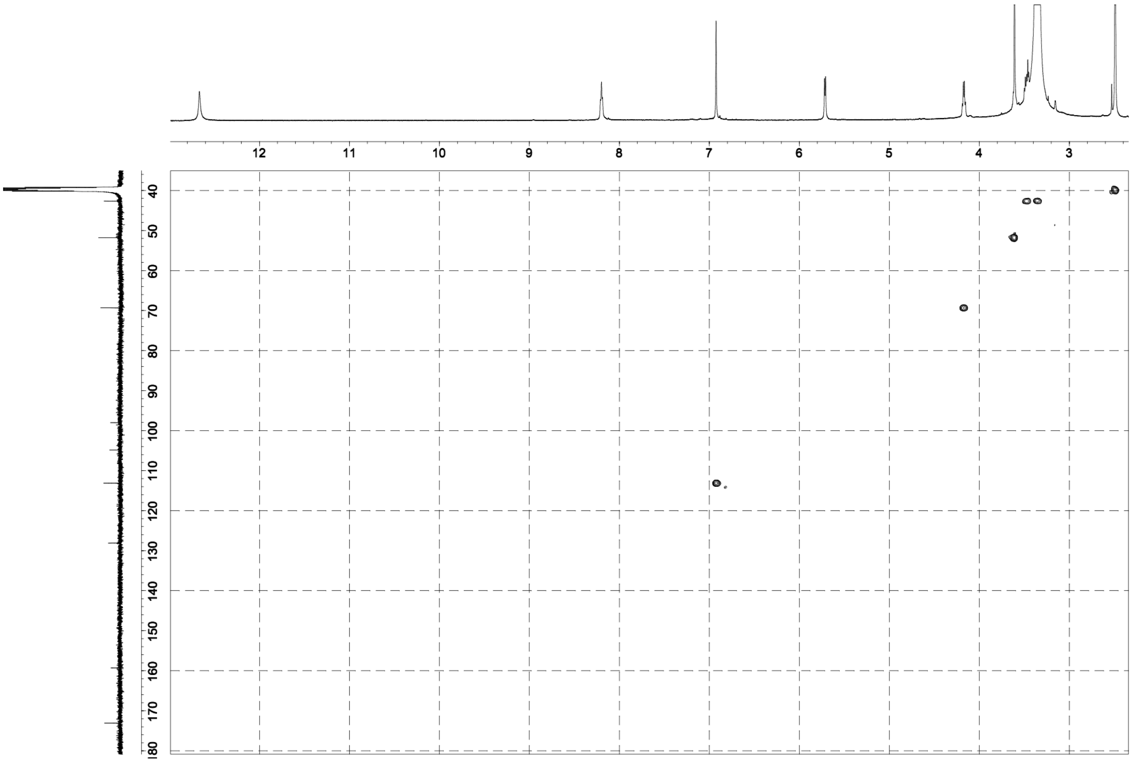

Figure S5. HMBC spectrum of agesasine A (**1**) in DMSO-d_6 (500 MHz).

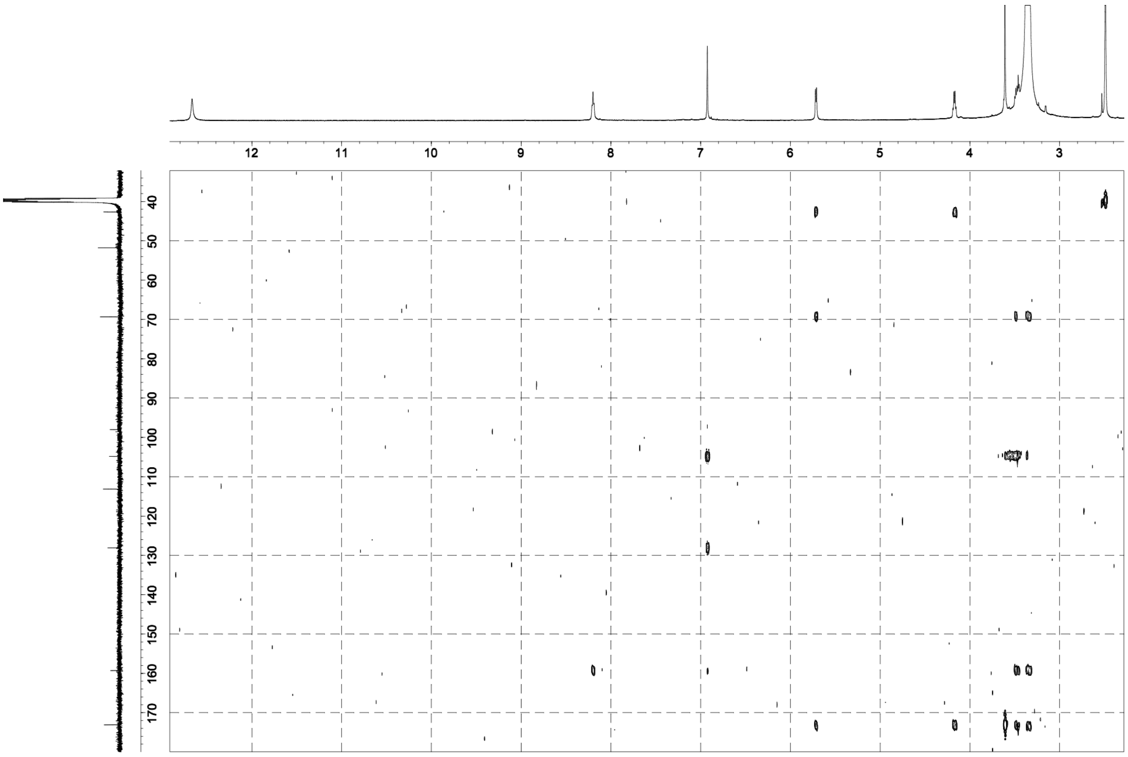

Figure S6. ROESY spectrum of agesasine A (**1**) in DMSO-d_6 (500 MHz).

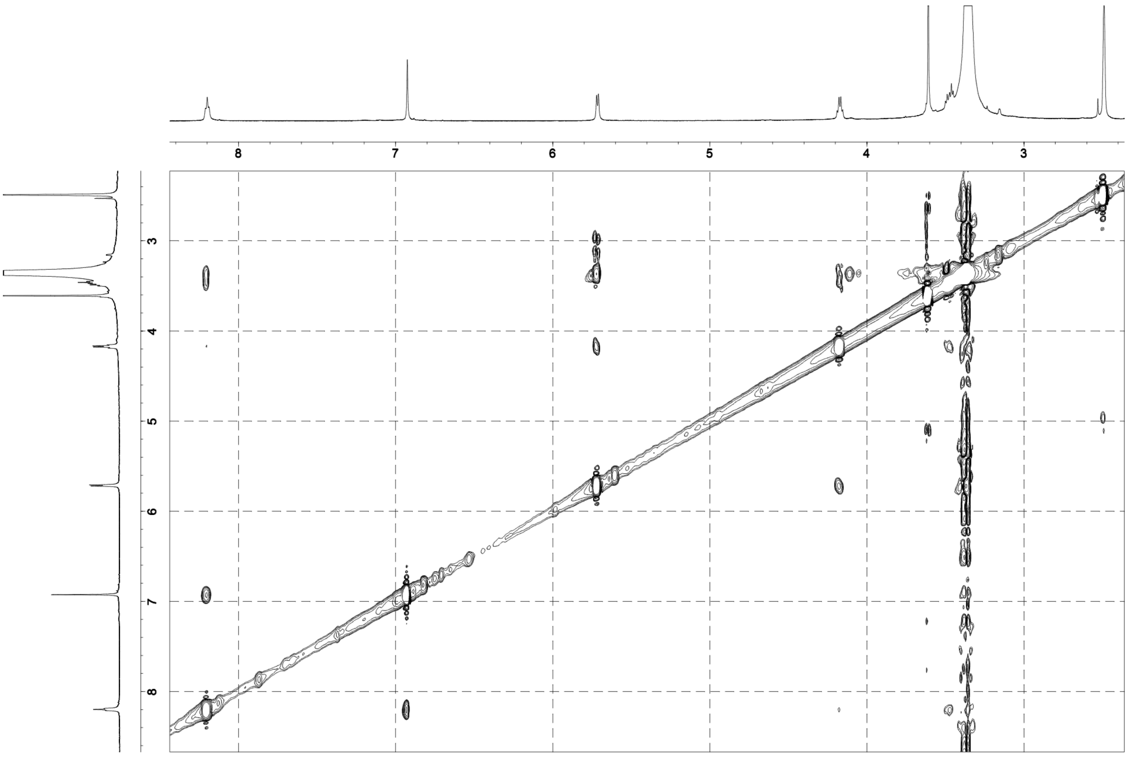

Figure S7. HRESIMS spectrum (pos.) of agesasine A (**1**).

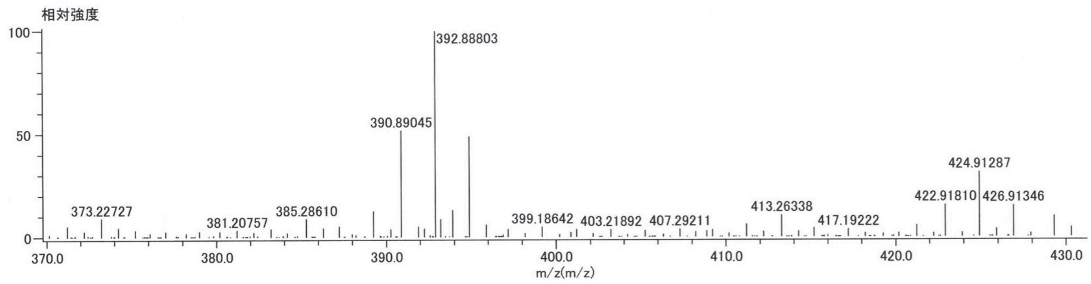

質量	強度	計算質量	質量差 mmu	質量差 ppm	推定組成式	不飽和数
390.89045	14874.61	390.89047	−0.01	−0.04	$^{12}C_{15}{}^{1}H_{7}{}^{79}Br_{1}{}^{81}Br_{1}{}^{14}N_{2}{}^{16}O_{1}$	12.5
		390.89050	−0.05	−0.13	$^{12}C_{9}{}^{1}H_{10}{}^{79}Br_{2}{}^{14}N_{2}{}^{23}Na_{1}{}^{16}O_{4}$	4.5
		390.89023	0.23	0.58	$^{12}C_{8}{}^{1}H_{11}{}^{79}Br_{2}{}^{14}N_{1}{}^{16}O_{7}$	3.0
		390.89015	0.30	0.77	$^{12}C_{8}{}^{1}H_{8}{}^{81}Br_{2}{}^{14}N_{5}{}^{23}Na_{1}{}^{16}O_{2}$	6.0
		390.89078	−0.33	−0.83	$^{12}C_{10}{}^{1}H_{18}{}^{79}Br_{3}{}^{16}O_{1}$	0.5

Figure S8. Chiral HPLC chart of agesasine A (**1**).

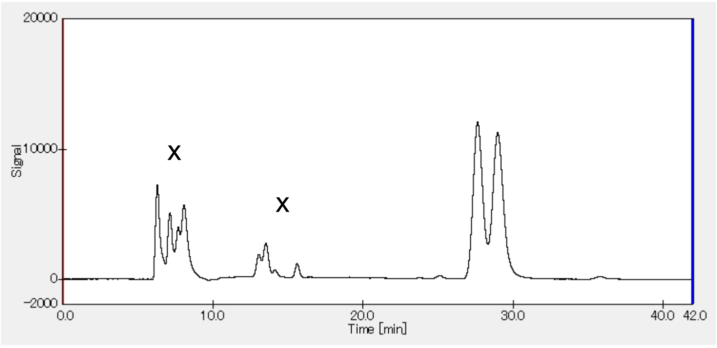

Figure S9. ^{1}H NMR spectrum of agesasine B (**2**) in DMSO-d_6 (500 MHz).

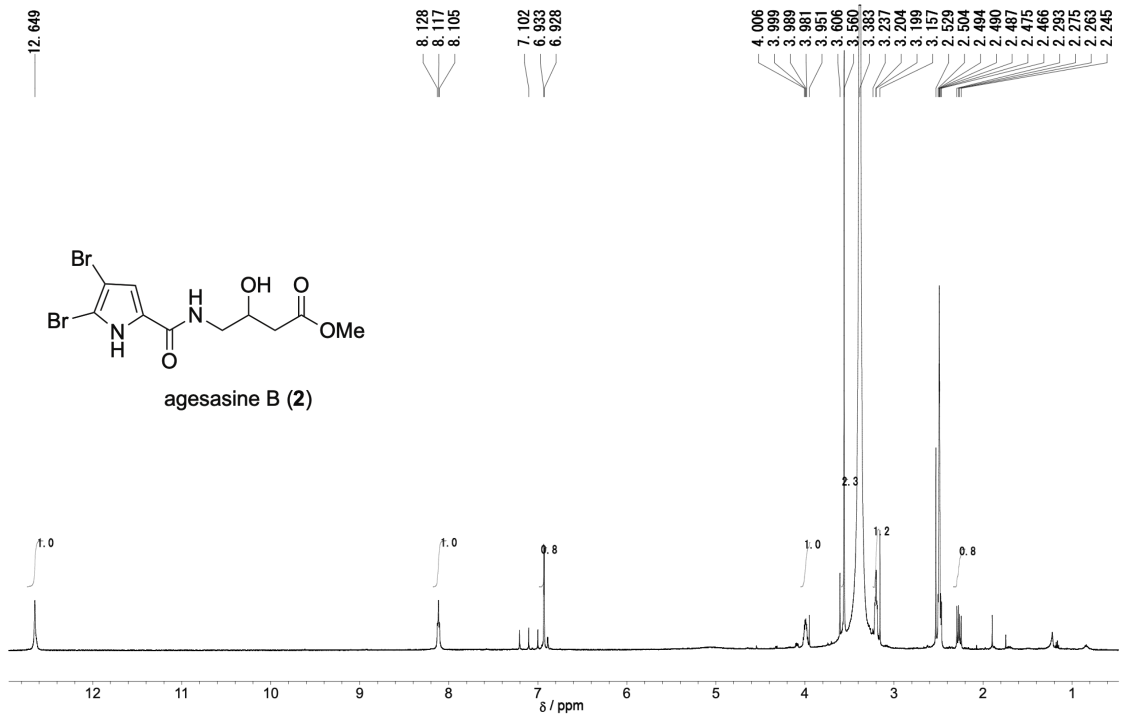

Figure S10. ^{13}C NMR spectrum of agesasine B (**2**) in DMSO-d_6 (125 MHz).

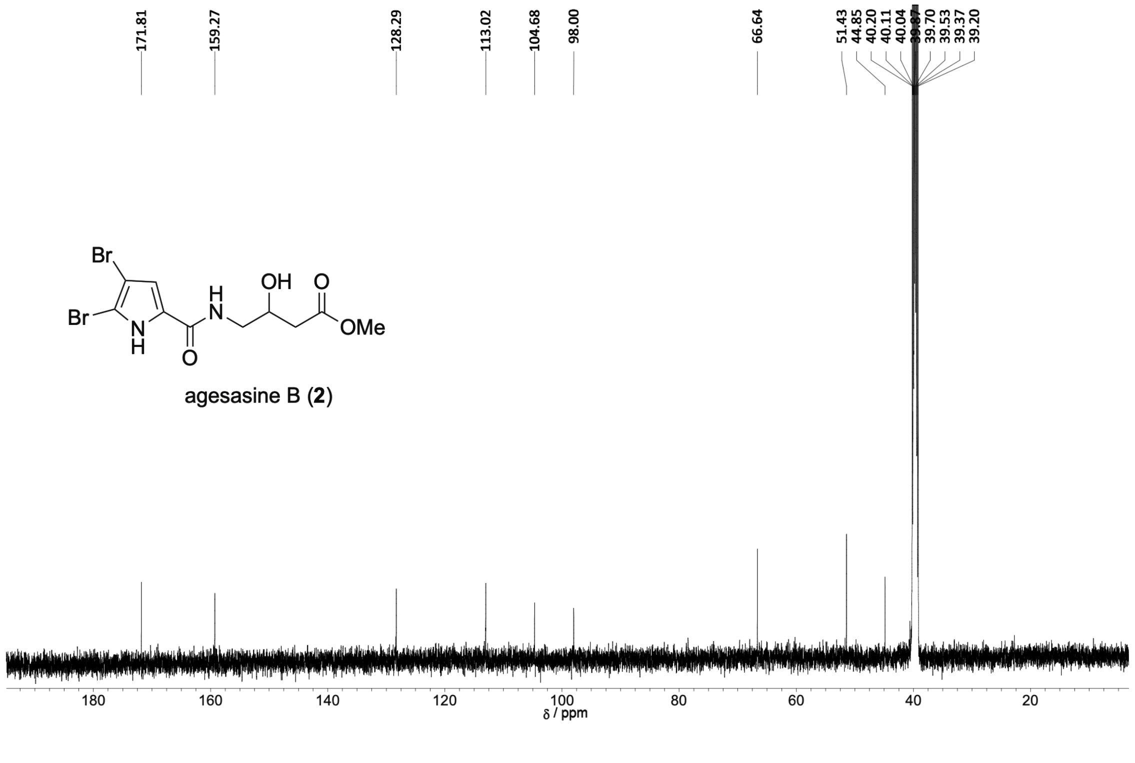

Figure S11. ^{1}H-^{1}H COSY spectrum of agesasine B (**2**) in DMSO-d_6 (500 MHz).

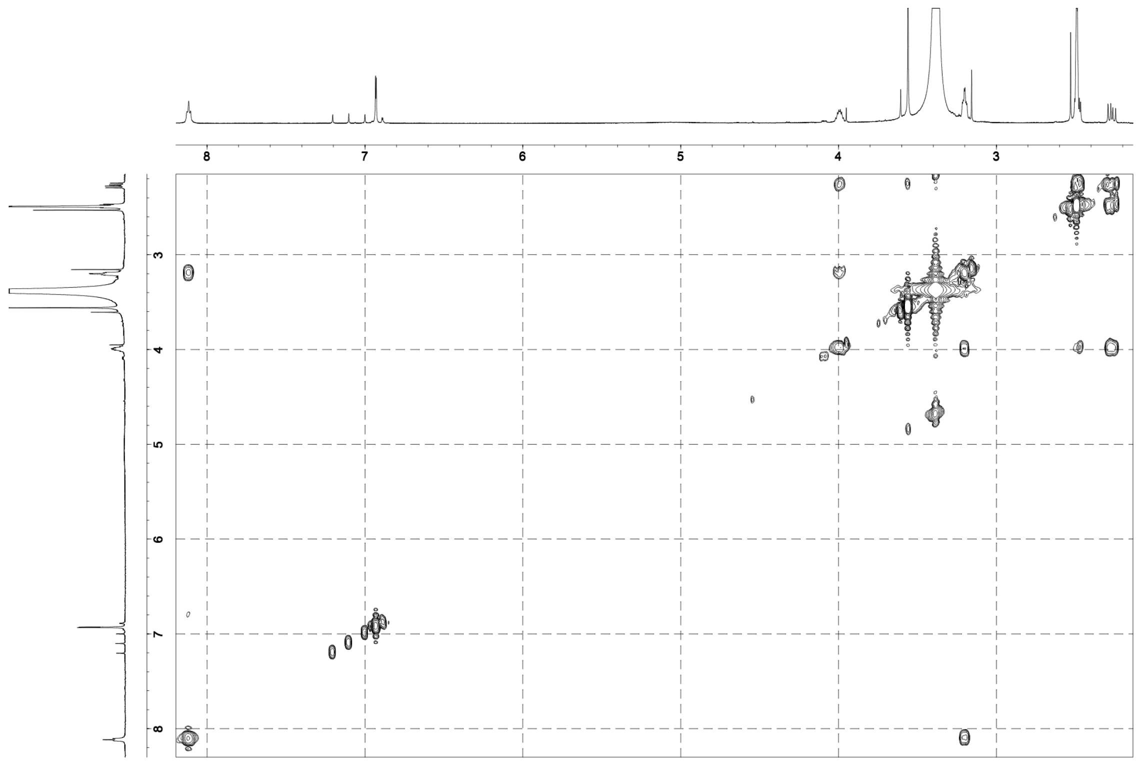

Figure S12. HSQC spectrum of agesasine B (**2**) in DMSO-d_6 (500 MHz).

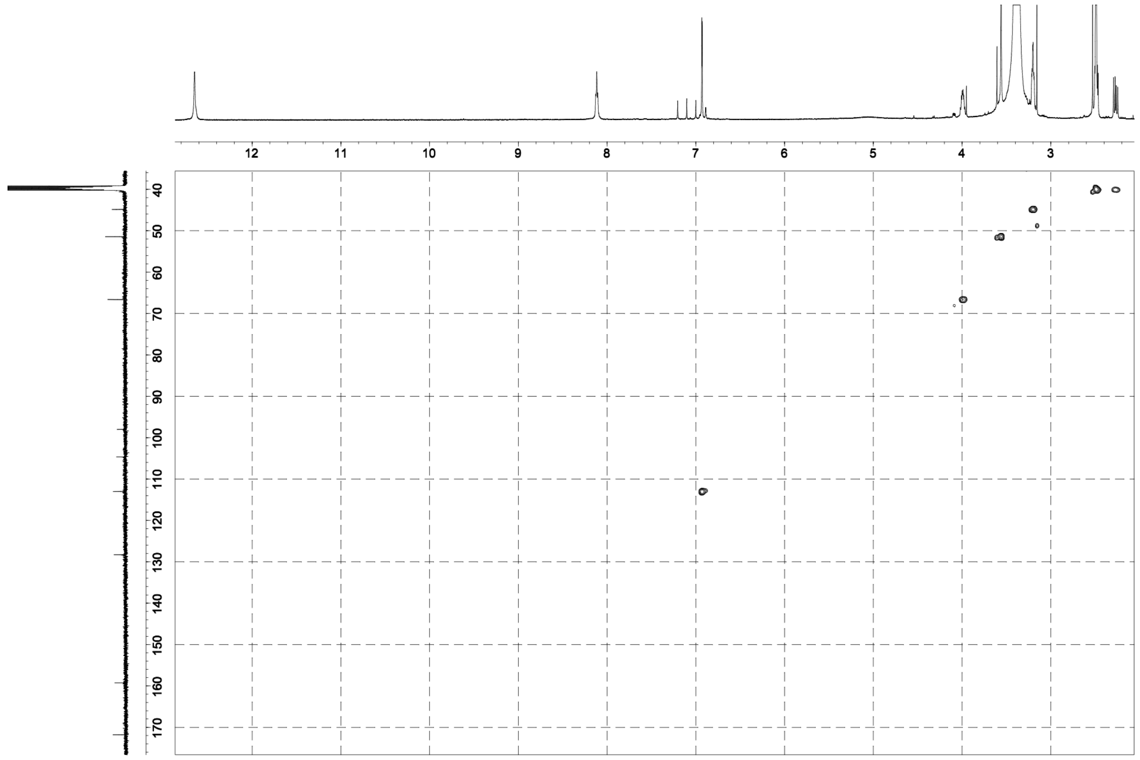

Figure S13. HMBC spectrum of agesasine B (**2**) in DMSO-d_6 (500 MHz).

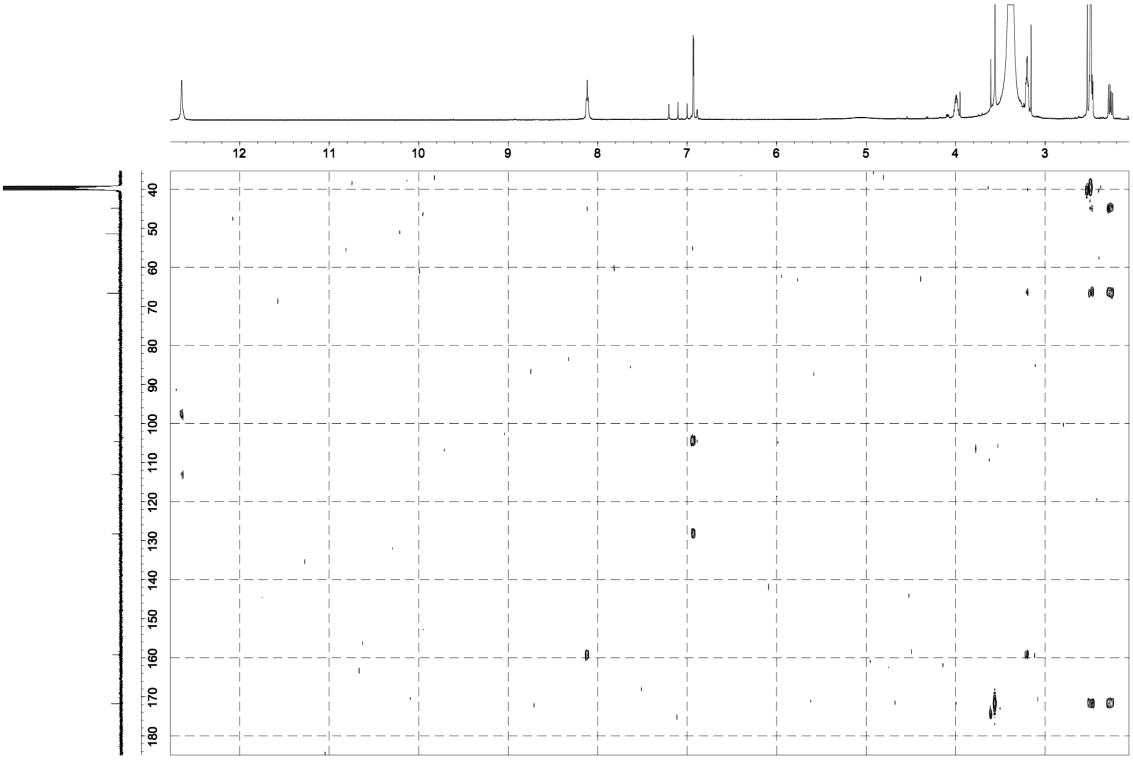

Figure S14. HRESIMS spectrum (neg.) of agesasine B (**2**).

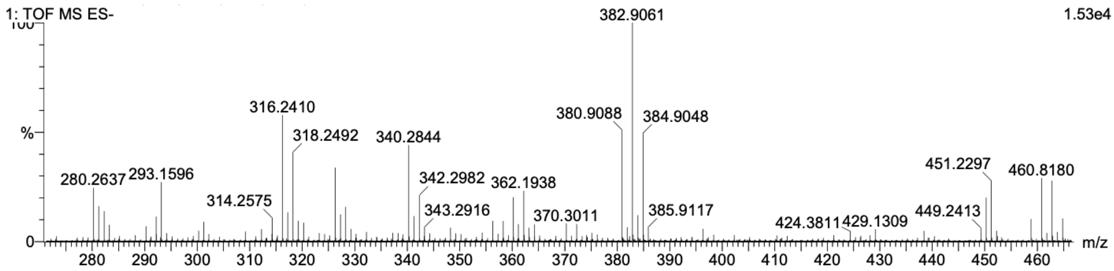

	Mass	RA	Calc. Mass	mDa	PPM	DBE	i-FIT	Formula
Minimum:		50.00				-1.5		
Maximum:		100.00		5.0	100.0	50.0		
	316.2410	57.74	---					
	380.9088	50.89	380.9086	0.2	0.5	5.5	26414954840064.0	C10 H11 N2 O4 79Br2
			380.8909	17.9	47.0	6.5	113240168202240.0	C10 H9 N2 O4 79Br 81Br
			380.8732	35.6	93.5	7.5	26609721540608.0	C10 H7 N2 O4 81Br2
	382.9061	100.00	382.9065	-0.4	-1.0	5.5	84645894946816.0	C10 H11 N2 O4 79Br 81Br
			382.8888	17.3	45.2	6.5	27109751783424.0	C10 H9 N2 O4 81Br2
			382.9242	-18.1	-47.3	4.5	26911325552640.0	C10 H13 N2 O4 79Br2

Figure S15. ^{1}H NMR spectrum of 9-hydroxydihydrodispacamide (**3**) in DMSO-d_6 (500 MHz).

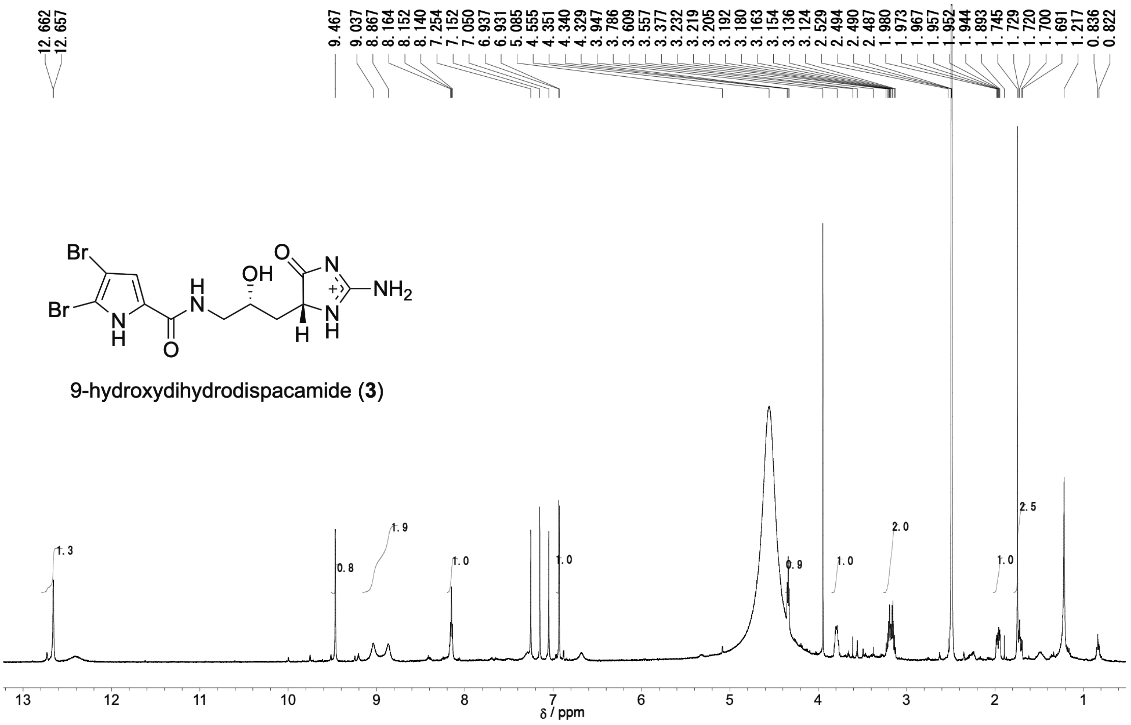

Figure S16. ^{13}C NMR spectrum of 9-hydroxydihydrodispacamide (**3**) in DMSO-d_6 (125 MHz).

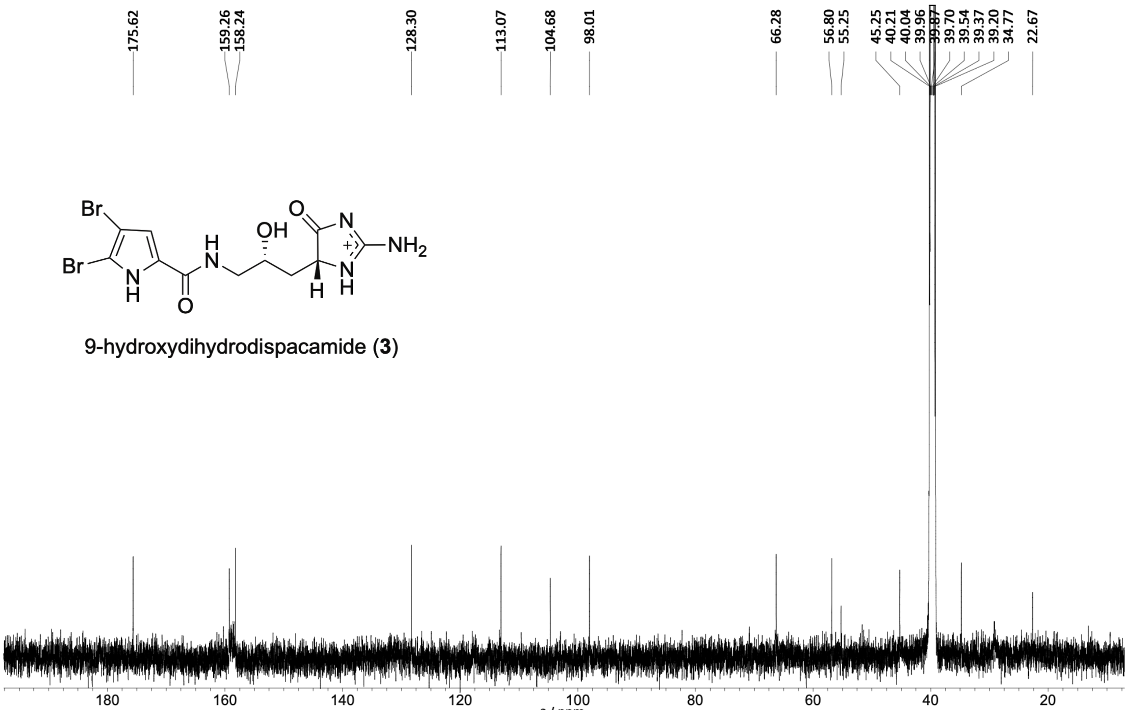

Figure S17. ^{1}H-^{1}H COSY spectrum of 9-hydroxydihydrodispacamide (**3**) in DMSO-d_6 (500 MHz).

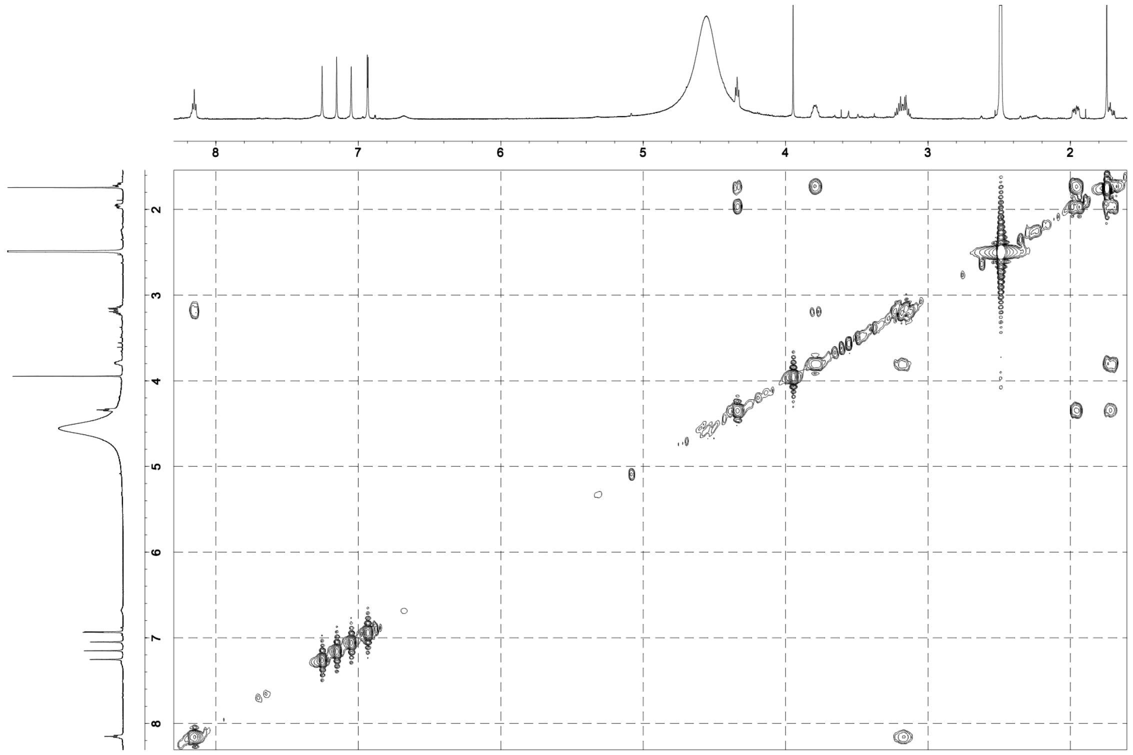

Figure S18. HSQC spectrum of 9-hydroxydihydrodispacamide (**3**) in DMSO-d_6 (500 MHz).

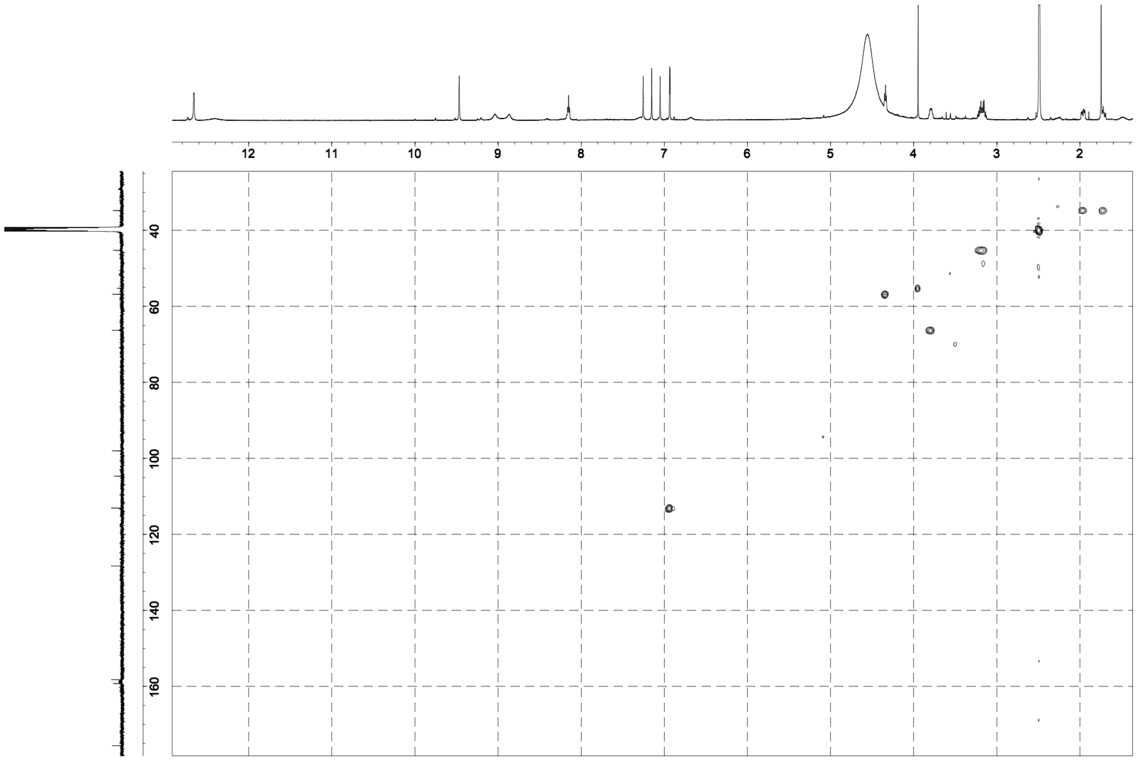

Figure S19. HMBC spectrum of 9-hydroxydihydrodispacamide (**3**) in DMSO-d_6 (500 MHz).

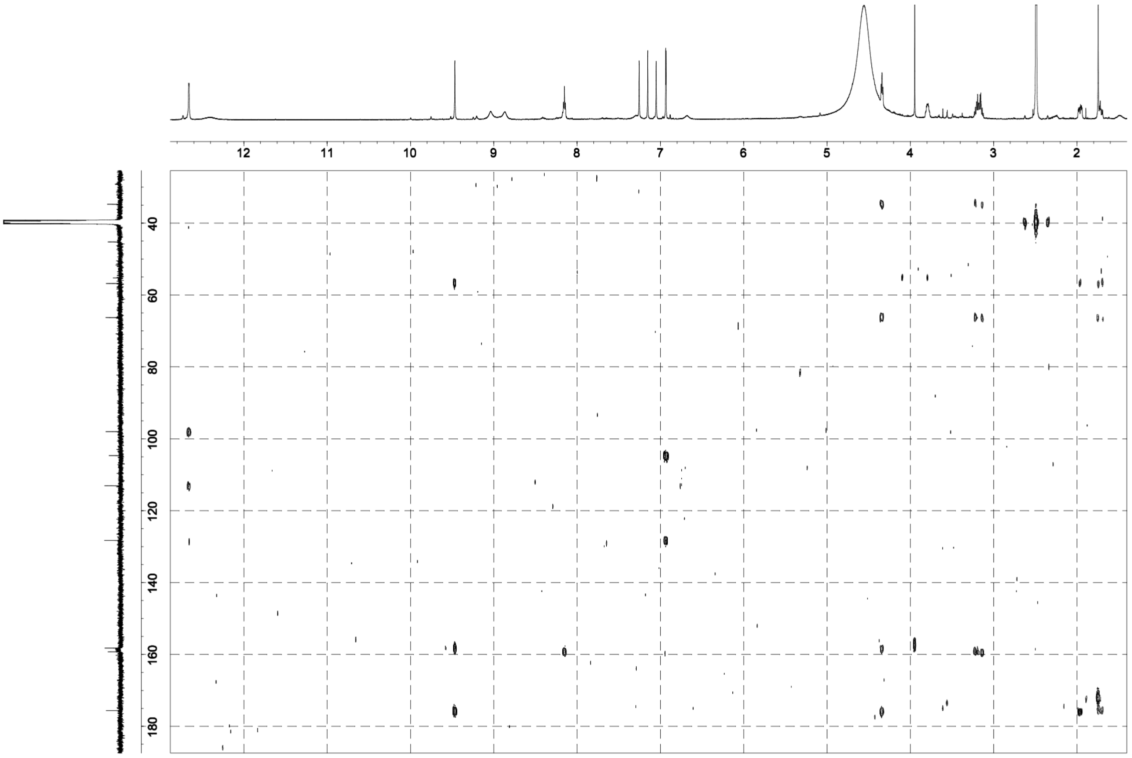

Figure S20. HRESIMS spectrum (pos.) of 9-hydroxydihydrodispacamide (**3**).

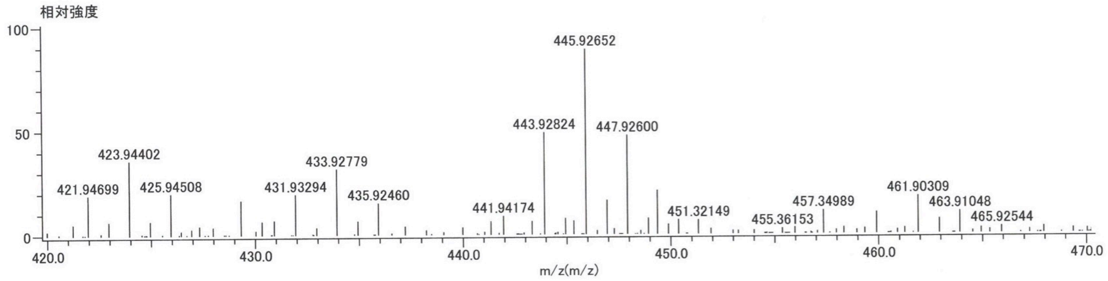

質量	強度	計算質量	質量差 mmu	質量差 ppm	推定組成式	不飽和数
443.92824	12999.25	443.92828	−0.04	−0.09	${}^{12}C_{11}{}^{1}H_{13}{}^{79}Br_{2}{}^{14}N_{5}{}^{23}Na_{1}{}^{16}O_{3}$	6.5
		443.92801	0.24	0.53	${}^{12}C_{10}{}^{1}H_{14}{}^{79}Br_{2}{}^{14}N_{4}{}^{16}O_{6}$	5.0
		443.92849	−0.25	−0.55	${}^{12}C_{13}{}^{1}H_{24}{}^{79}Br_{1}{}^{81}Br_{2}{}^{23}Na_{1}$	0.0
		443.92794	0.31	0.69	${}^{12}C_{11}{}^{1}H_{17}{}^{81}Br_{2}{}^{14}N_{1}{}^{23}Na_{1}{}^{16}O_{6}$	2.5
		443.92793	0.31	0.70	${}^{12}C_{10}{}^{1}H_{11}{}^{81}Br_{2}{}^{14}N_{8}{}^{23}Na_{1}{}^{16}O_{1}$	8.0
		443.92856	−0.32	−0.71	${}^{12}C_{12}{}^{1}H_{21}{}^{79}Br_{3}{}^{14}N_{3}$	2.5
		443.92773	0.51	1.15	${}^{12}C_{9}{}^{1}H_{6}{}^{79}Br_{1}{}^{14}N_{6}{}^{23}Na_{1}{}^{16}O_{9}$	9.0
		443.92770	0.55	1.23	${}^{12}C_{15}{}^{1}H_{3}{}^{81}Br_{1}{}^{14}N_{6}{}^{16}O_{6}$	17.0
		443.92769	0.55	1.24	${}^{12}C_{3}{}^{1}H_{15}{}^{79}Br_{1}{}^{81}Br_{1}{}^{14}N_{7}{}^{23}Na_{1}{}^{16}O_{7}$	−1.5
		443.92766	0.58	1.31	${}^{12}C_{10}{}^{1}H_{18}{}^{81}Br_{2}{}^{16}O_{9}$	1.0
		443.92766	0.59	1.32	${}^{12}C_{9}{}^{1}H_{12}{}^{81}Br_{2}{}^{14}N_{7}{}^{16}O_{4}$	6.5
		443.92750	0.75	1.68	${}^{12}C_{12}{}^{1}H_{24}{}^{79}Br_{3}{}^{23}Na_{1}{}^{16}O_{1}$	−1.0
		443.92900	−0.76	−1.71	${}^{12}C_{11}{}^{1}H_{14}{}^{81}Br_{2}{}^{14}N_{4}{}^{16}O_{5}$	6.0
		443.92908	−0.83	−1.87	${}^{12}C_{11}{}^{1}H_{8}{}^{79}Br_{1}{}^{14}N_{3}{}^{23}Na_{1}{}^{16}O_{10}$	8.5
		443.92928	−1.03	−2.32	${}^{12}C_{12}{}^{1}H_{13}{}^{81}Br_{2}{}^{14}N_{5}{}^{23}Na_{1}{}^{16}O_{2}$	7.5

Figure S21. Chiral HPLC chart of 9-hydroxydihydrodispacamide (**3**).

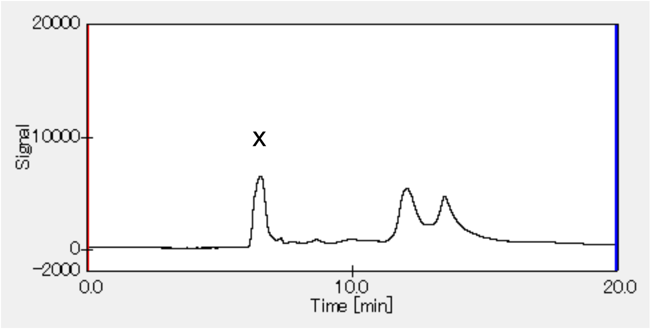

Figure S22. ^{1}H NMR spectrum of 9-hydroxydihydrooroidin (**4**) in DMSO-d_6 (500 MHz).

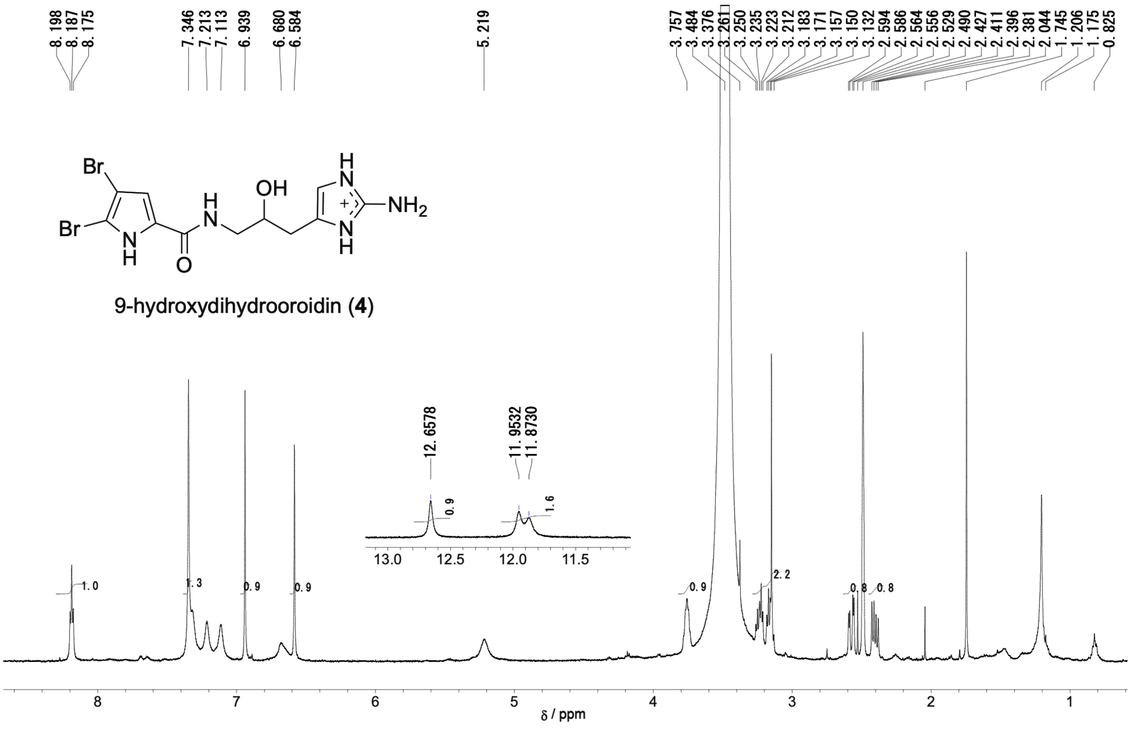

Figure S23. ^{13}C NMR spectrum of 9-hydroxydihydrooroidin (**4**) in DMSO-d_6 (125 MHz).

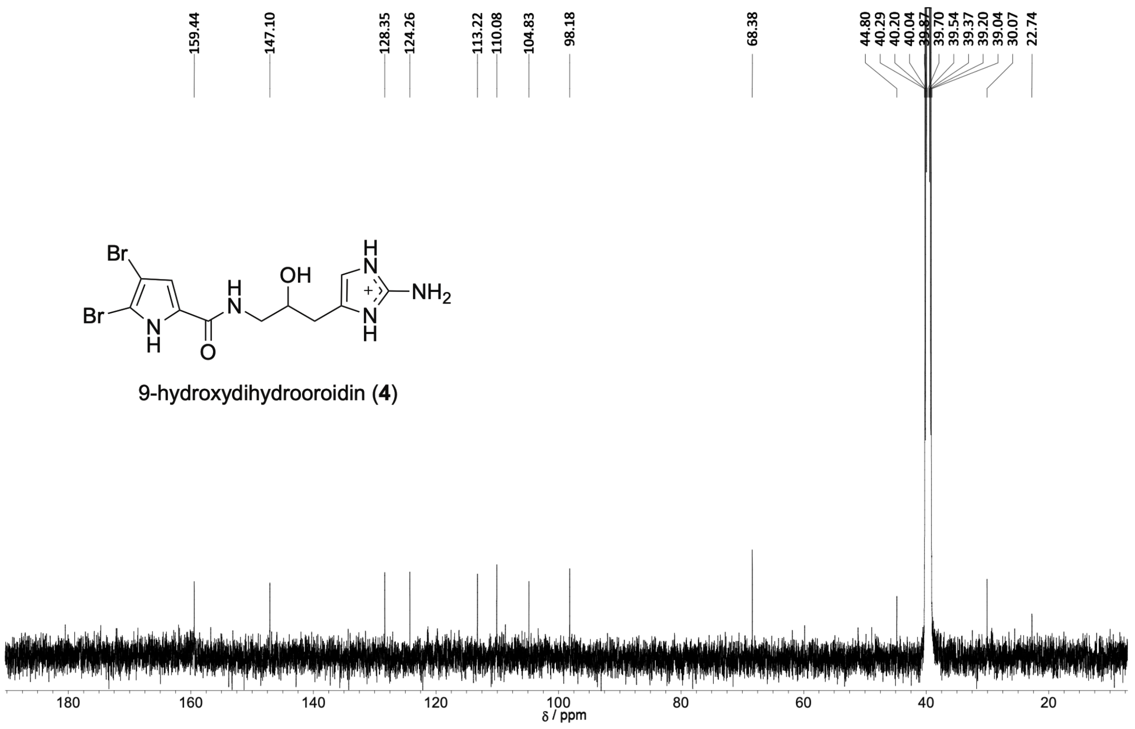

Figure S24. ^{1}H-^{1}H COSY spectrum of 9-hydroxydihydrooroidin (**4**) in DMSO-d_6 (500 MHz).

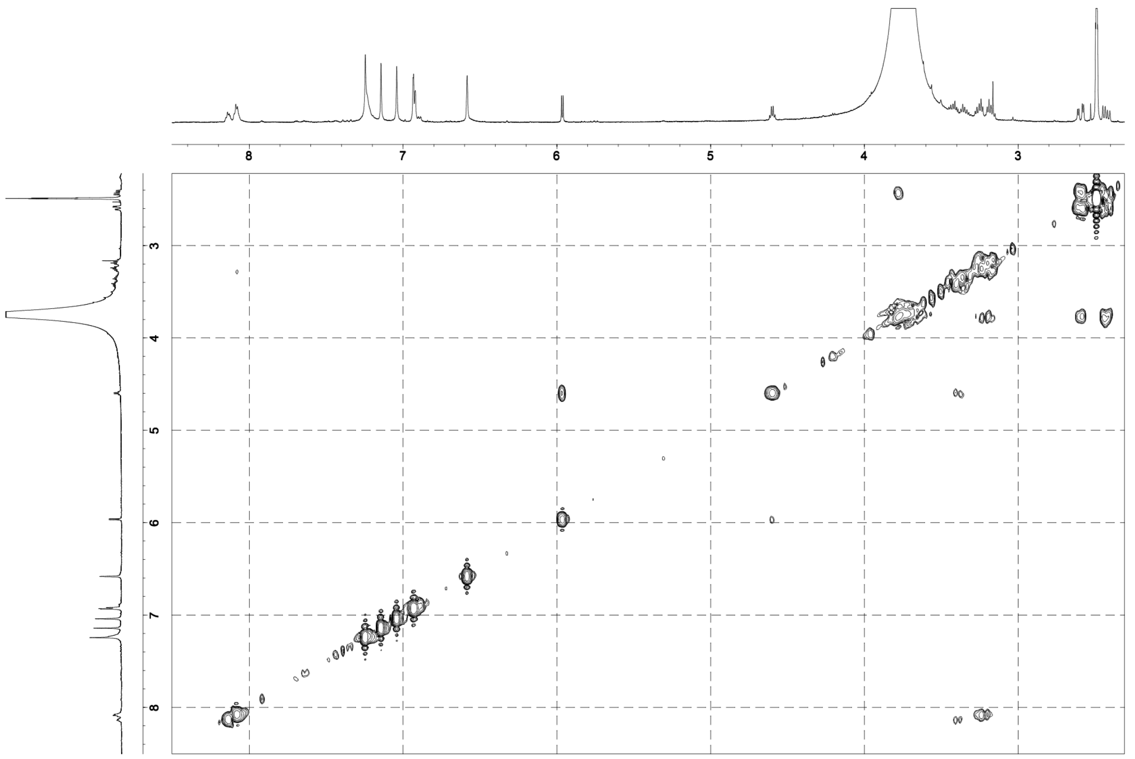

Figure S25. HSQC spectrum of 9-hydroxydihydrooroidin (**4**) in DMSO-d_6 (500 MHz).

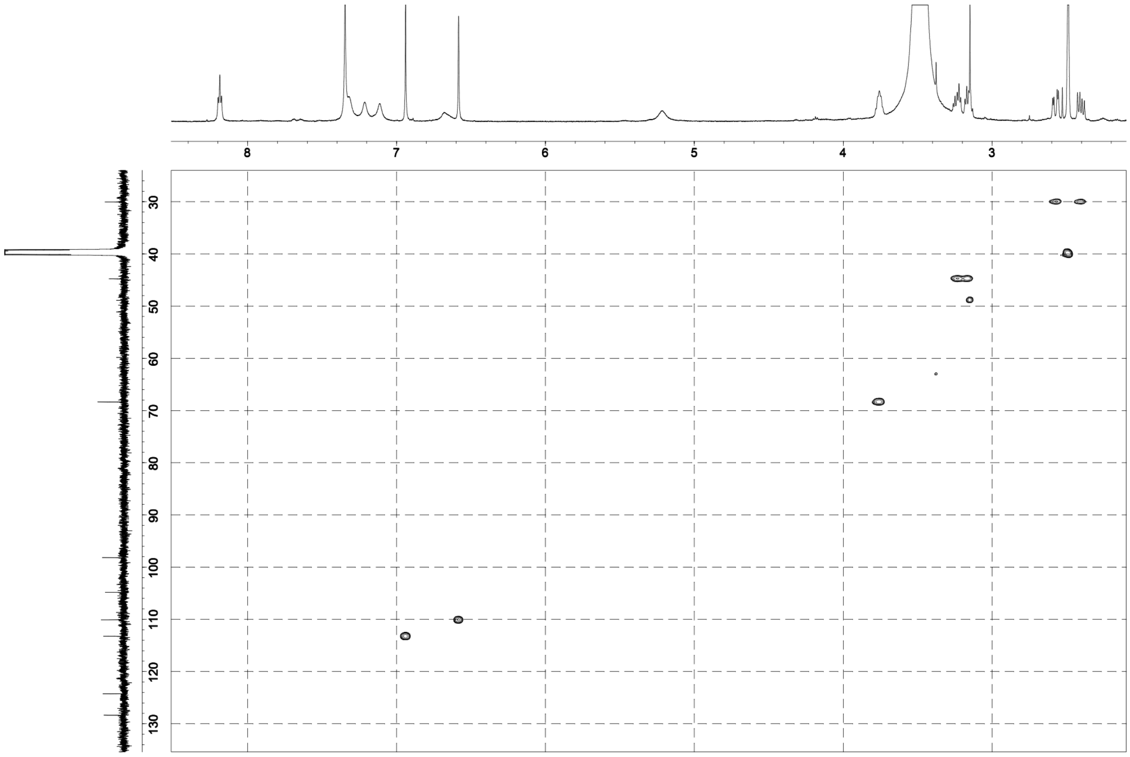

Figure S26. HMBC spectrum of 9-hydroxydihydrooroidin (**4**) in DMSO-d_6 (500 MHz).

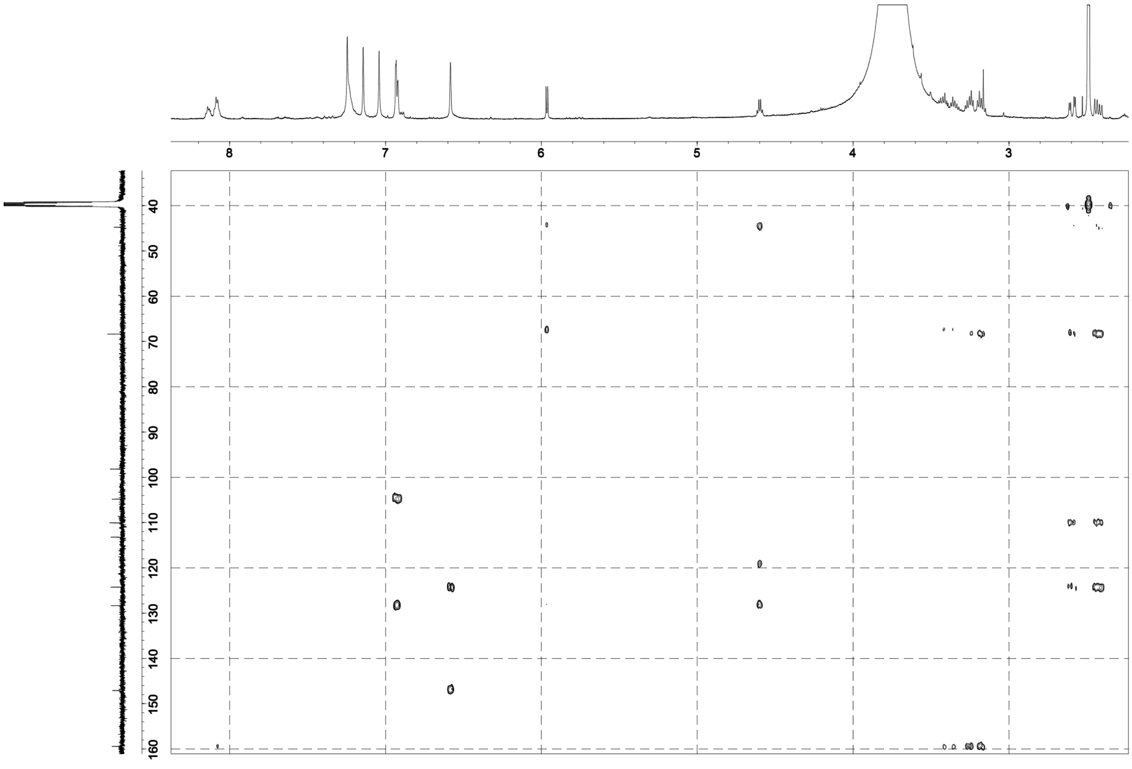

Figure S27. HRESIMS spectrum (pos.) of 9-hydroxydihydrooroidin (**4**).

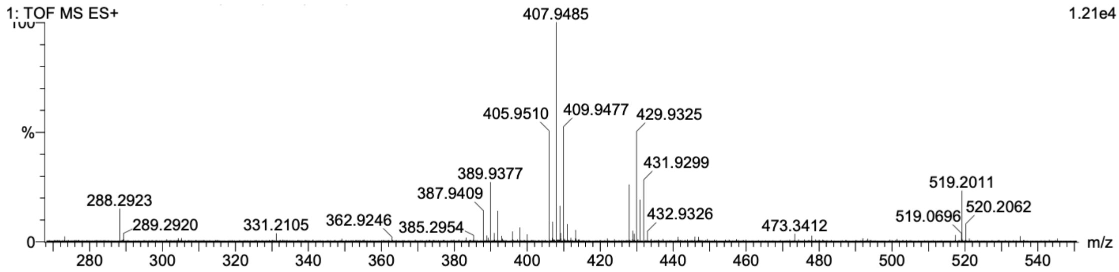

Minimum:	50.00				-1.5		
Maximum:	100.00		5.0	100.0	50.0		
Mass	RA	Calc. Mass	mDa	PPM	DBE	i-FIT	Formula
405.9510	50.52	405.9514	-0.4	-1.0	6.5	6333292085248.0	C11 H14 N5 O2 79Br2
407.9485	100.00	---					
409.9477	52.30	---					
429.9325	50.20	429.9490	-16.5	-38.4	5.5	566160064512.0	C11 H15 N5 O2 Na 79Br2

Figure S28. ECD spectrum of 9-hydroxydihydrooroidin (**4**) in MeOH.

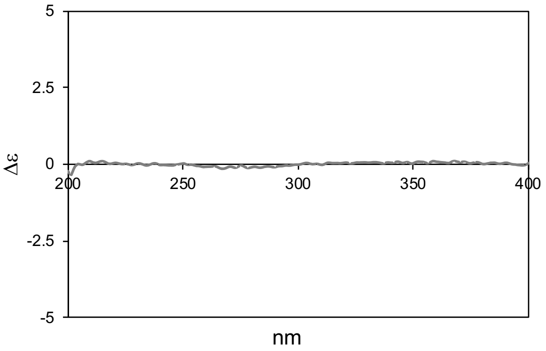

Figure S29. ^{1}H NMR spectrum of 9*E*-keramadine (**5**) in DMSO-d_6 (500 MHz).

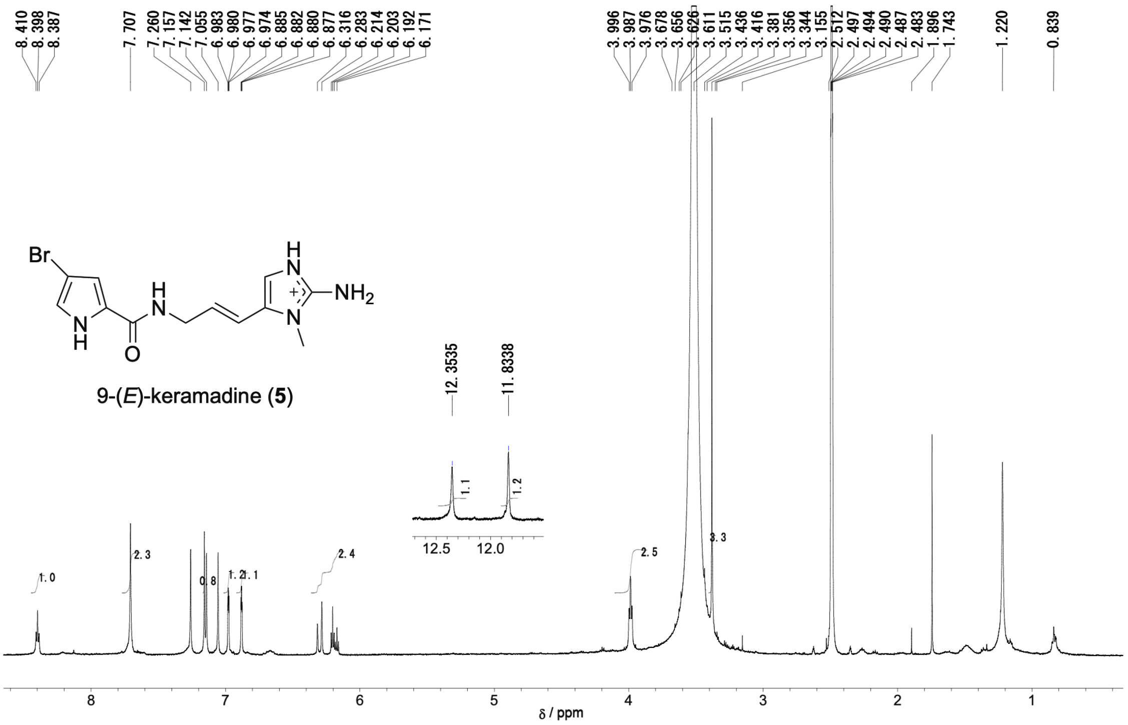

Figure S30. ^{13}C NMR spectrum of 9*E*-keramadine (**5**) in DMSO-d_6 (125 MHz).

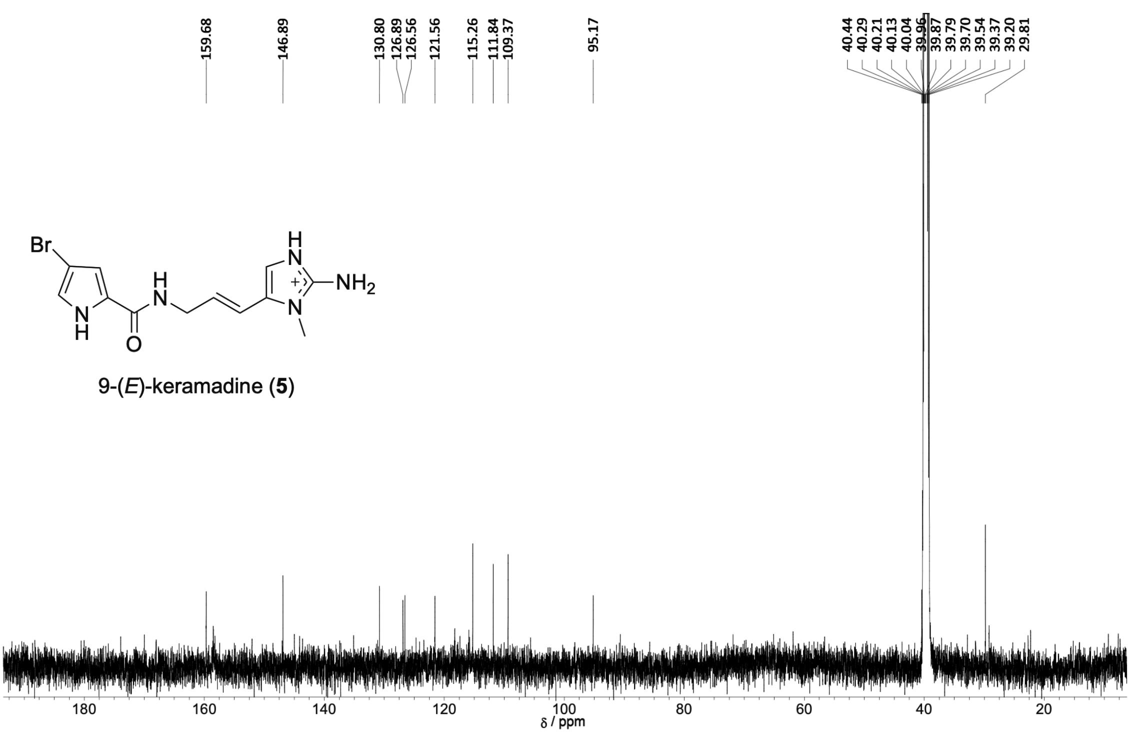

Figure S31. ^{1}H-^{1}H COSY spectrum of 9*E* -keramadine (**5**) in DMSO-d_6 (500 MHz).

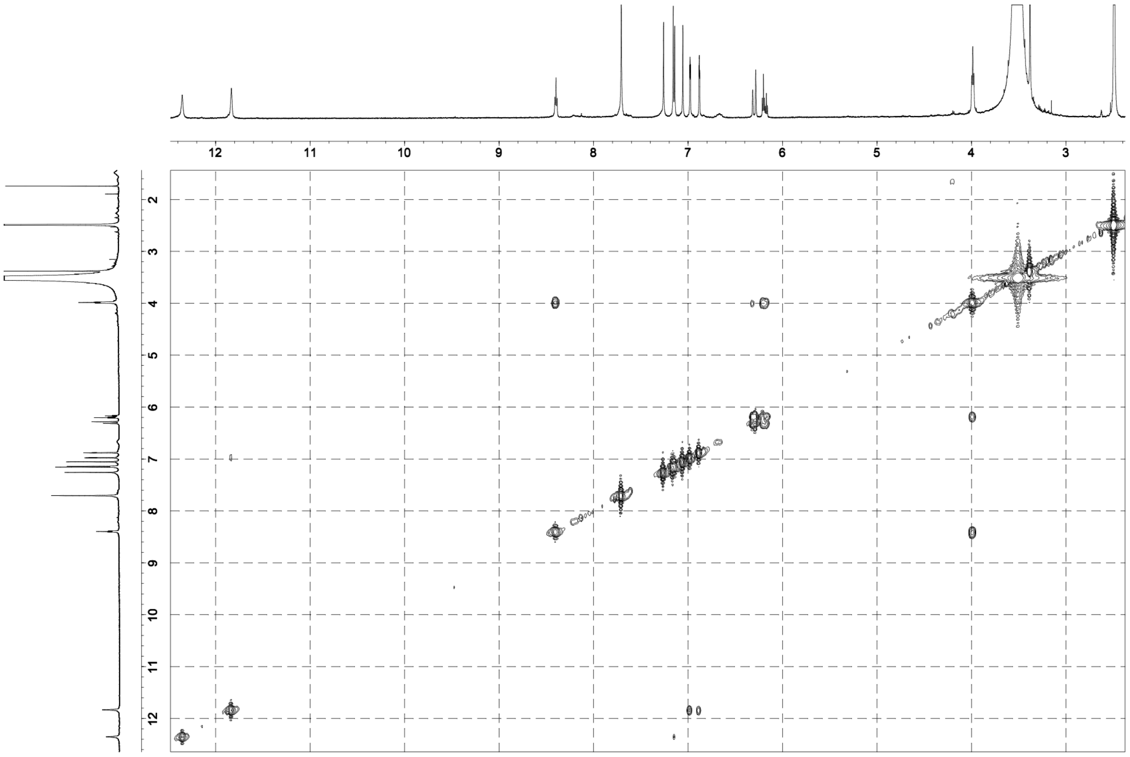

Figure S32. HSQC spectrum of 9*E* -keramadine (**5**) in DMSO-d_6 (500 MHz).

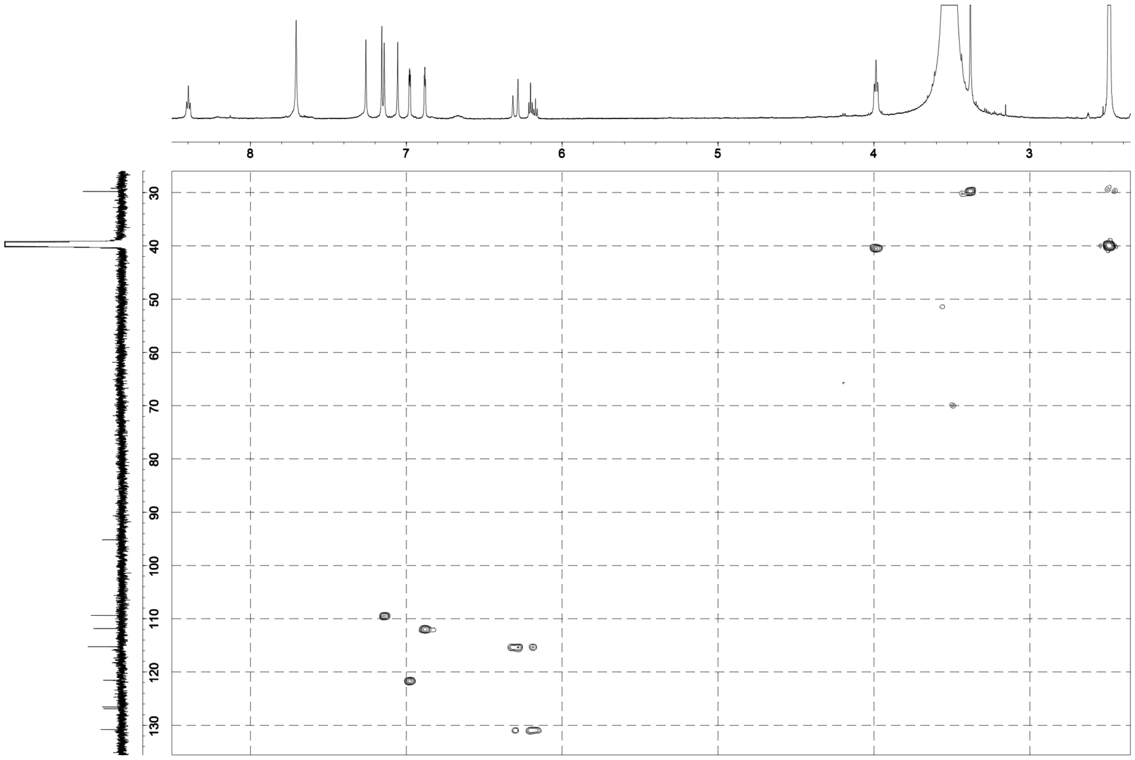

Figure S33. HMBC spectrum of 9*E* -keramadine (**5**) in DMSO-d_6 (500 MHz).

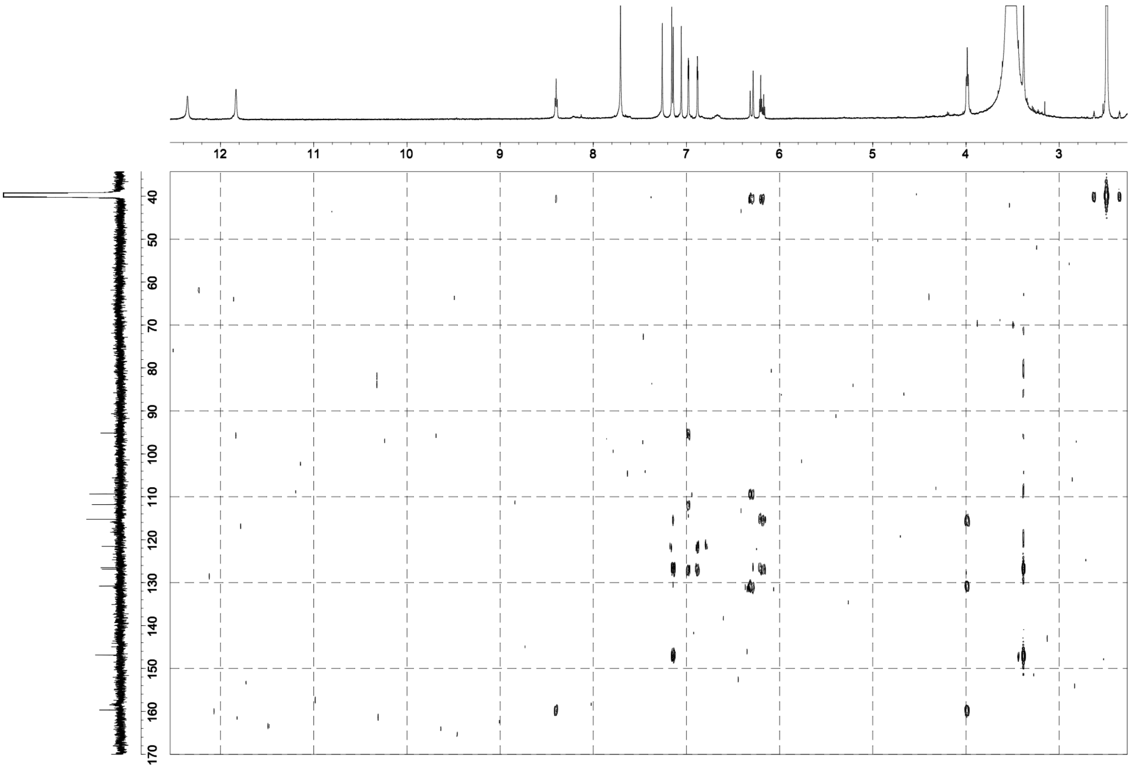

Figure S34. ROESY spectrum of 9*E* -keramadine (**5**) in DMSO-d_6 (500 MHz).

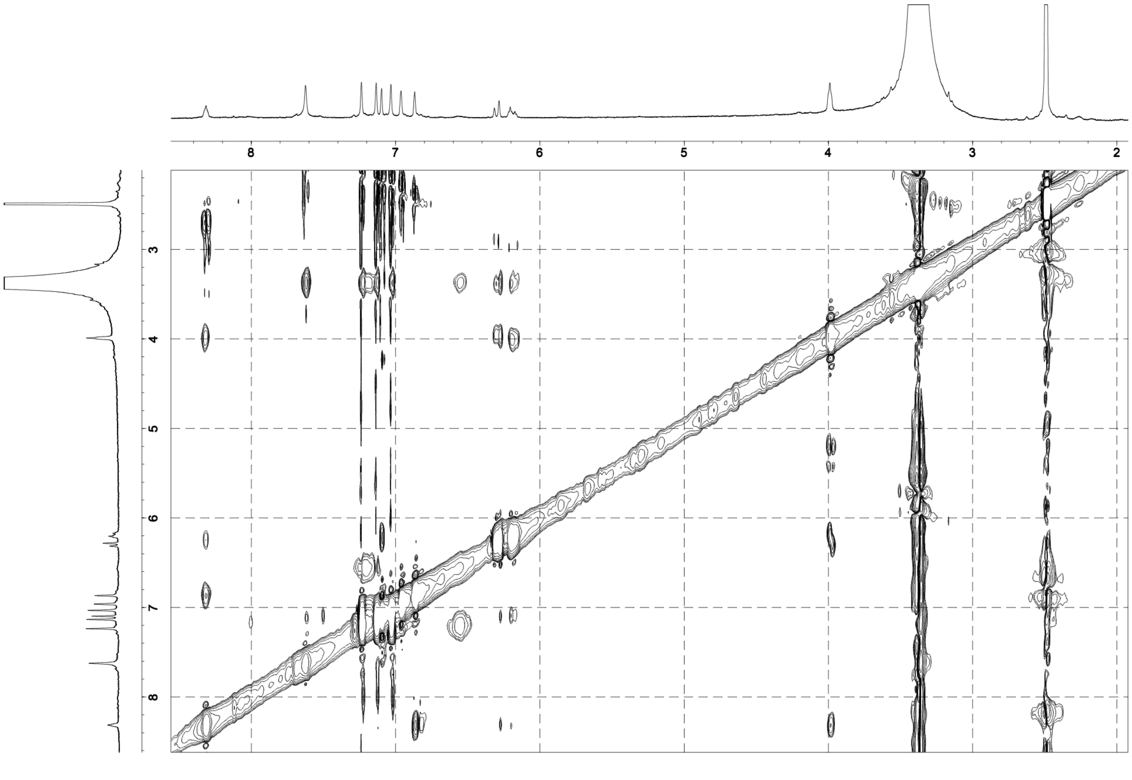

Figure S35. HRESIMS spectrum (pos.) of 9-(*E*)-keramadine (**5**).

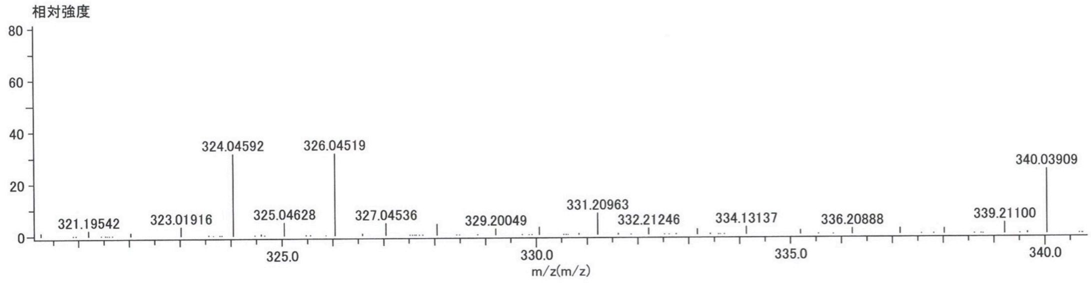

質量	強度	計算質量	質量差 mmu	質量差 ppm	推定組成式	不飽和数
324.04592	9847.05	324.04600	−0.08	−0.24	$^{12}C_{12}{}^{1}H_{15}{}^{79}Br_{1}{}^{14}N_{5}{}^{16}O_{1}$	7.5
		324.04573	0.19	0.60	$^{12}C_{12}{}^{1}H_{13}{}^{23}Na_{1}{}^{16}O_{9}$	6.0
		324.04572	0.20	0.61	$^{12}C_{11}{}^{1}H_{7}{}^{14}N_{7}{}^{23}Na_{1}{}^{16}O_{4}$	11.5
		324.04568	0.24	0.73	$^{12}C_{5}{}^{1}H_{16}{}^{81}Br_{1}{}^{14}N_{8}{}^{23}Na_{1}{}^{16}O_{2}$	1.0

Figure S36. ^{1}H NMR spectrum of tauroacidin A in DMSO-d_6 (500 MHz).

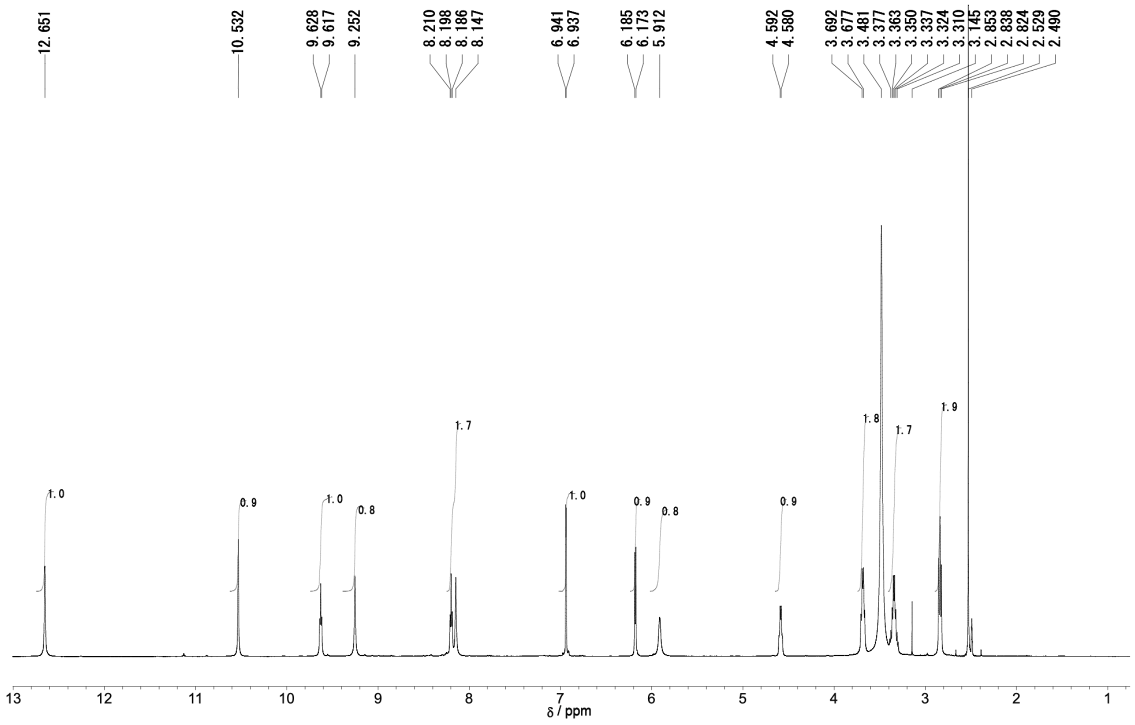

Figure S37. ^{1}H NMR spectrum of taurodispacamide A in DMSO-d_6 (500 MHz).

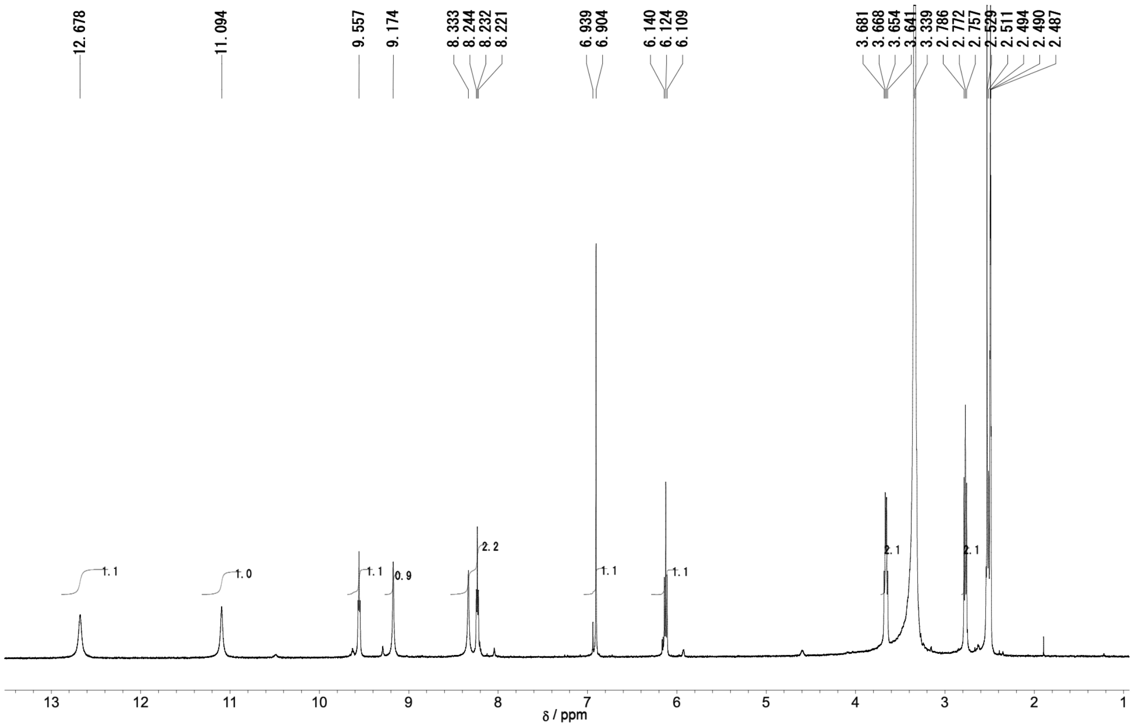

Figure S38. ^{1}H NMR spectrum of oroidin in DMSO-d_6 (500 MHz).

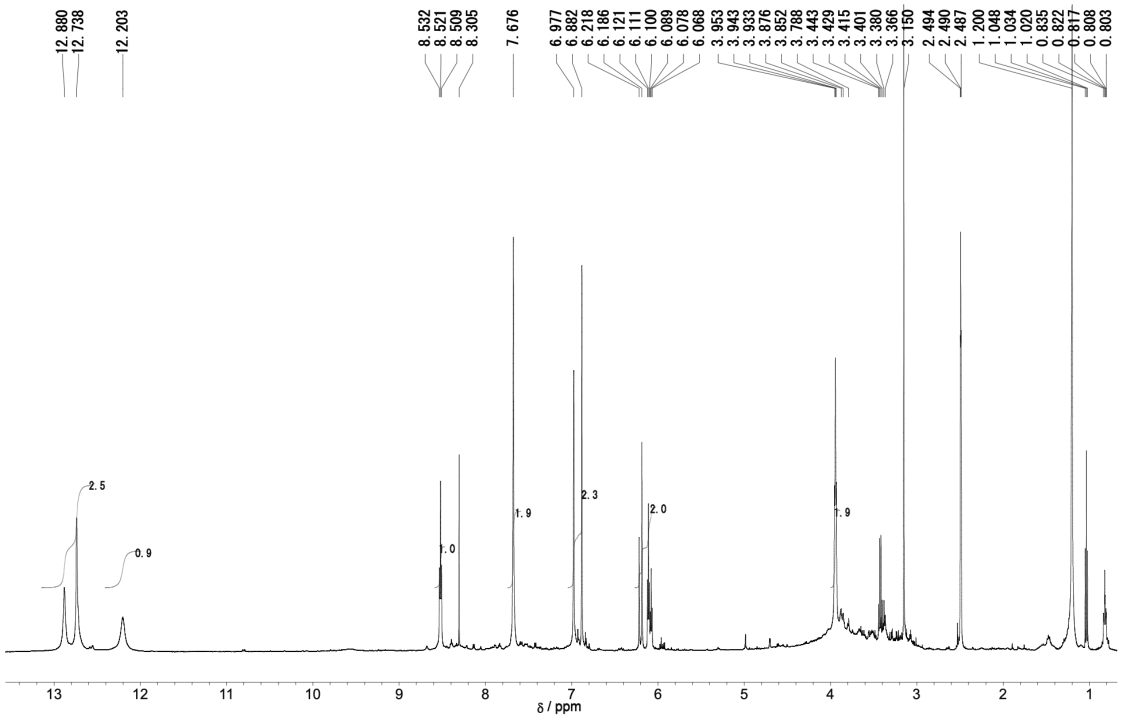

Figure S39. ^{1}H NMR spectrum of keramadine in DMSO-d_6 (500 MHz).

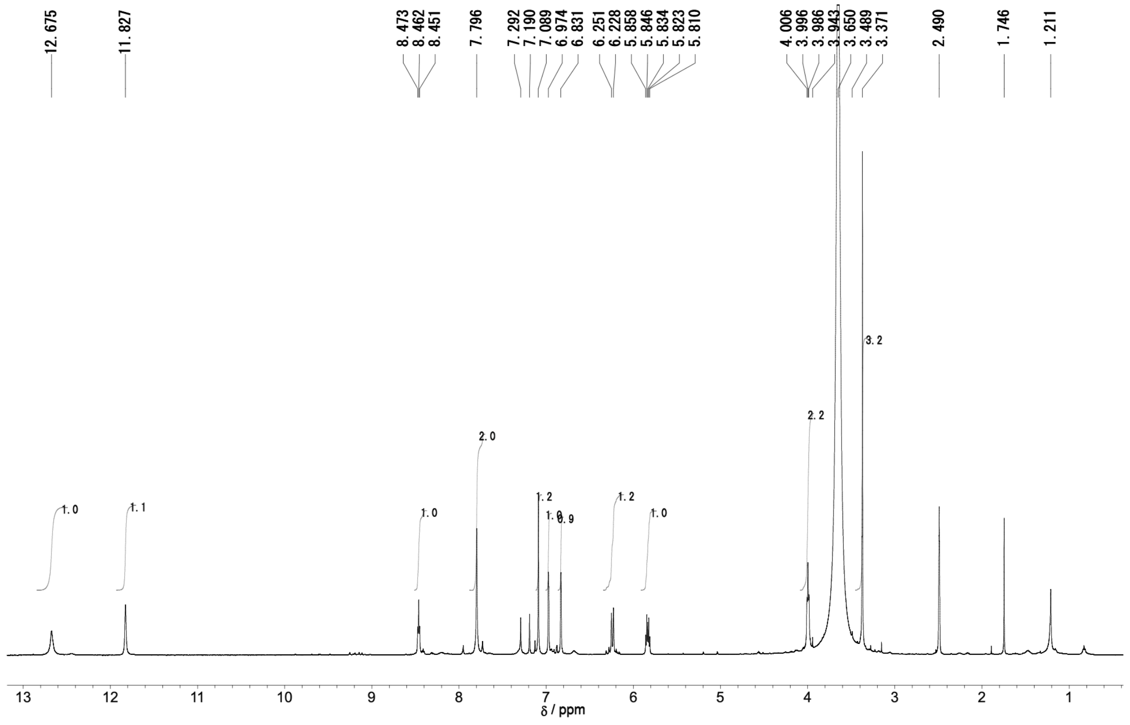

Figure S40. ^{1}H NMR spectrum of 2-bromo-9,10-dihydrokeramadine in DMSO-d_6 (500 MHz).

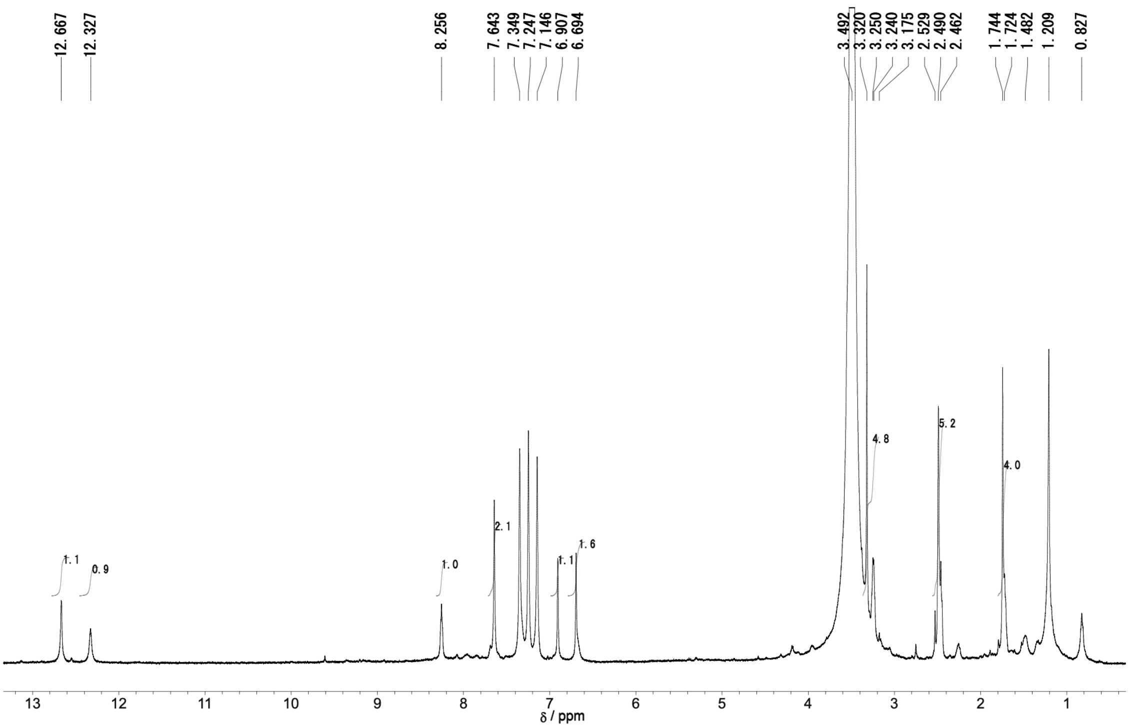

Figure S41. ¹H NMR spectrum of nagelamide L in DMSO-d_6 (500 MHz).

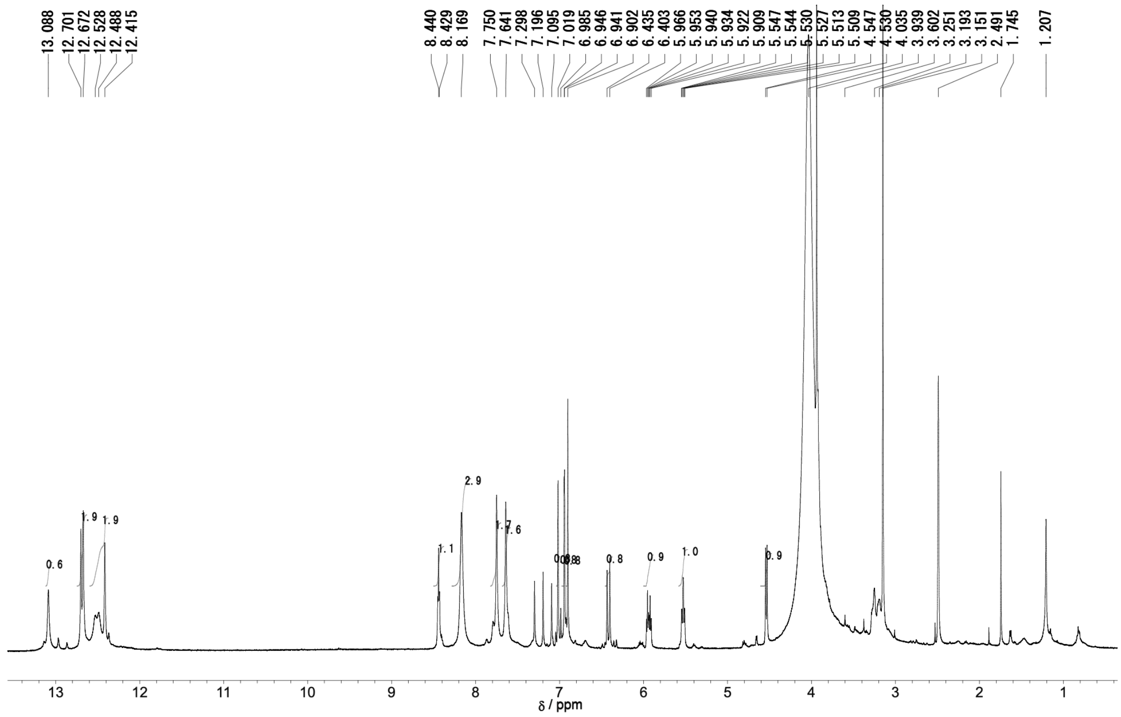

Figure S42. Structures of known bromopyrrole alkaloids, tauroacidin A, taurodispacamide A, oroidin, keramadine, 2-bromokeramadine, and nagelamide L.

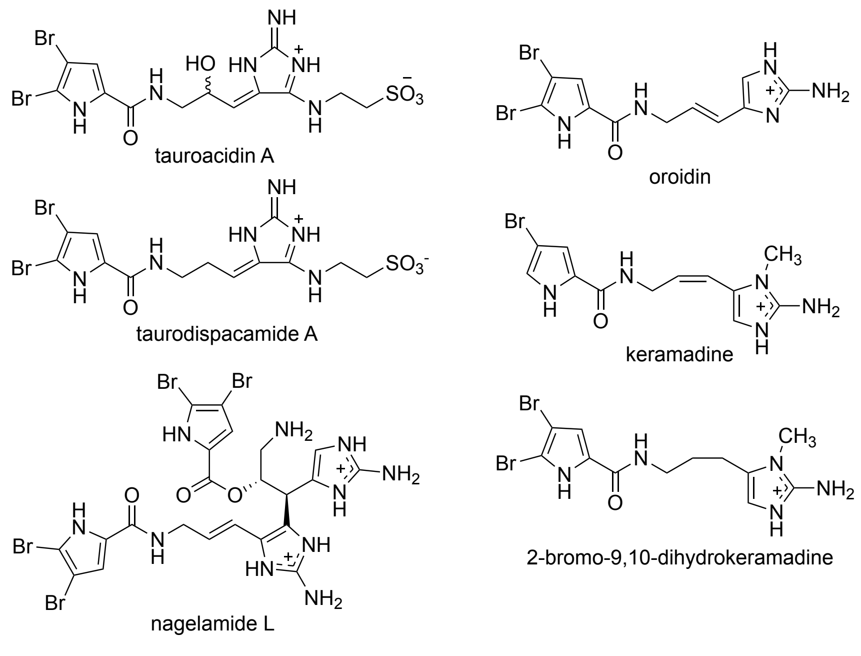

Figure S43. Antiproliferative activity of **1**–**5** against HeLa cells.

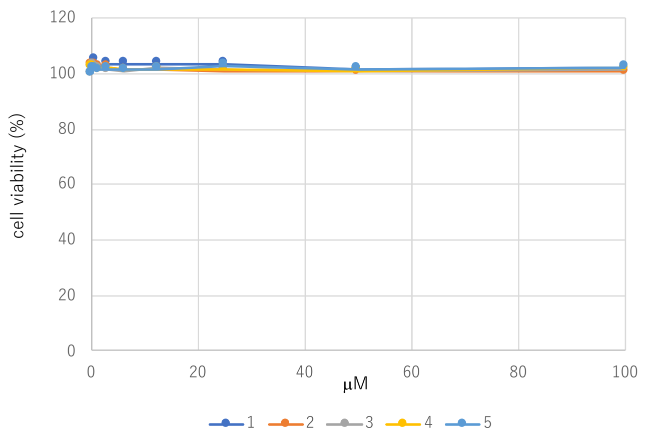

Figure S44. Antiproliferative activity of **1**–**5** against A549 cells.

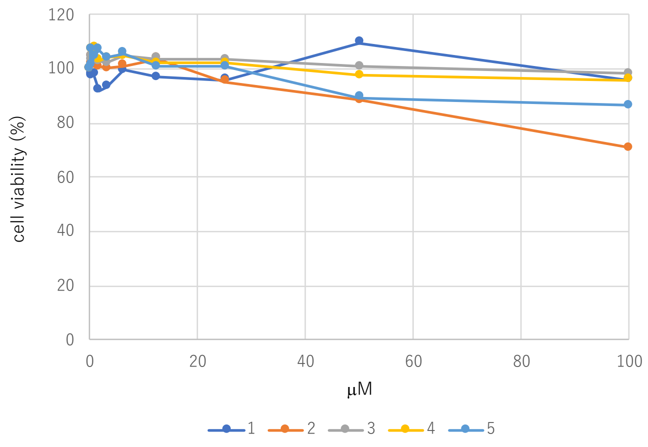

Figure S41. Antiproliferative activity of **1**–**5** against MCF7 cells.

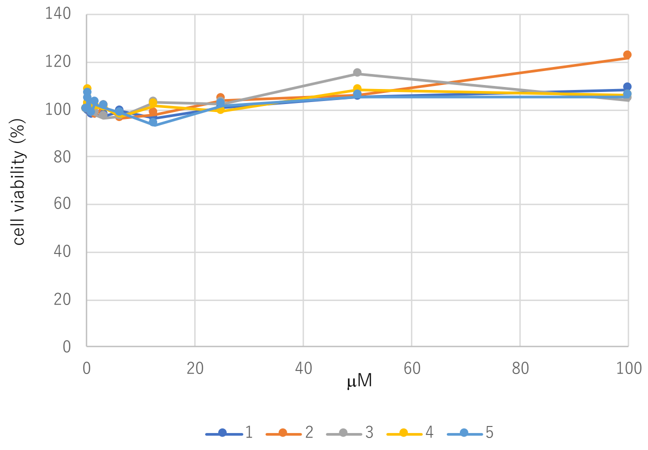

Table S1. 1D and 2D NMR data for agesasine A (**1**) in DMSO-d_6.

position	^{13}C	^{1}H (*J* in Hz)	^{1}H-^{1}H COSY	HMBC (*H* to *C*)	ROESY
1	–	12.67 (brs)			
2	104.8	–			
3	98.0	–			
4	113.1	6.93 (brs)		2, 5, 6	
5	128.1	–			
6	159.3	–			
7	–	8.20 (t, 5.8)	8	6	4, 8
8	42.7	3.46, 3.36 (each 1H, m)	7, 9	9, 6, 10	7, 9, 9-OH
9	69.3	4.17 (q, 6.1)	8, 9-OH	8, 10	8, 9-OH
10	173.1	–			
9-OH		5.71 (d, 5.9)	9	8, 9, 10	8, 9
OMe	51.8	3.61 (3H, brs)		10	

Table S2. 1D and 2D NMR data for agesasine B (**2**) in DMSO-d_6.

position	^{13}C	^{1}H (*J* in Hz)	^{1}H-^{1}H COSY	HMBC (*H* to *C*)
1	–	12.65 (brs)		3, 4
2	104.7	–		
3	98.0	–		
4	113.0	6.93 (d, 2,7)		2, 5
5	128.3	–		
6	159.3	–		
7	–	8.12 (t, 5.5)	8	6
8	44.9	3.20 (2H, m)	7, 9	
9	66.6	3.99 (m)	8, 10	
10	40.6	2.49 (m), 2.27 (dd, 15.2, 8.8)	9	8, 9, 11
11	171.8			
9-OH		*nd*		
OMe	51.4	3.56 (3H, brs)		11

nd: not detected

Table S3. 1D and 2D NMR data for 9-hydroxydihydrodispacamide (**3**) in DMSO-d_6.

position	^{13}C	^{1}H (J in Hz)	^{1}H-^{1}H COSY	HMBC (H to C)
1	–	12.66 (brs)		3, 4, 5
2	104.7	–		
3	98.0	–		
4	113.1	6.94 (d, 2.8)		2, 5
5	128.3	–		
6	159.3	–		
7	–	8.15 (t, 5.9)	8	6
8	45.3	3.18 (2H, m)	7, 9	6, 9, 10
9	66.3	3.79 (m)	8, 10	
10	34.8	1.96 (ddd, 14.4, 5.5, 2.6)	9, 11	9, 11, 15
		1.71 (ddd, 14.4, 10.9, 5.5)		
11	56.8	4.34 (t, 5.5)	10	9, 10, 13, 15
12	–	9.47 (brs)		11, 13, 15
13	158.2	–		
14	–	*nd*		
15	175.6	–		
13-NH_2	–	*nd*		

nd: not detected

Table S4. 1D and 2D NMR data for 9-hydroxydihydrooroidin (**4**) in DMSO-d_6.

position	^{13}C	^{1}H (*J* in Hz)	^{1}H-^{1}H COSY	HMBC (*H* to *C*)
1	–	12.66 (brs)		
2	104.8	–		
3	98.2	–		
4	113.2	6.86 (s)		2, 5
5	128.4	–		
6	159.4	–		
7	–	8.19 (t, 5.6)	8	6
8	44.8	3.23 (m), 3.16 (m)	7, 9	6, 9
9	68.4	3.76 (m)	8, 10	
10	30.1	2.57 (dd, 15.2, 4.2)<br>2.40 (dd, 15.2, 7.8)	9	9, 11, 15
11	124.3	–		
12	–	11.95 (brs)		
13	147.1	–		
14	–	11.87 (brs)		
15	110.1	6.58 (brs)		11, 13
13-NH_2	–	7.35 (2H, brs)		

Figure S5. 1D and 2D NMR data for 9-(*E*)-keramadine (**5**) in DMSO-d_6.

position	^{13}C	^{1}H (*J* in Hz)	^{1}H-^{1}H COSY	HMBC (*H* to *C*)	ROESY
1	–	11.83 (brs)	2, 4		
2	121.6	6.98 (dd, 2.9, 1.6)	1	3, 4, 5	
3	95.2	–			
4	111.8	6.92 (s)		2, 5	
5	126.9	–			
6	159.7	–			
7	–	8.40 (t, 5.5)	8	6	2, 8
8	40.4	3.99 (2H, t, 5.5)	7, 9, 10	6, 9, 10	10
9	130.8	6.19 (dt, 16.1, 5.5)	8, 10	8, 10, 11	15
10	115.3	6.30 (d, 16.1)	8, 9	8, 9, 15	8, N-Me
11	126.6	–			
13	146.9	–			
14	–	12.35 (brs)			
15	109.4	7.14 (brs)		11, 13	9
N-Me	29.8	3.38 (3H, s)			10
13-NH_2	–	7.71 (2H, brs)			

Table S6. ^{1}H NMR data for tauroacidin A and taurodispacamide A in DMSO-d_6 (500 MHz).

position	**tauroacidin A**	**taurodispacamide A**
	^{1}H (J in Hz)	^{1}H (J in Hz)
1	12.65 (brs)	12.68 (brs)
4	6.94 (d, 2.4)	6.90 (s)
7	8.20 (t, 6.1)	8.23 (t, 5.5)
8	3.34 (2H, m)	3.34 (2H, m)[a]
9	4.59 (q, 5.8)	2.51 (2H, m)
10	6.18 (d, 5.8)	6.12 (t, 7.7)
12	10.53 (1H, brs)	11.09 (s)
14	9.25, (brs)	9.17 (brs)
1’	3.69 (2H, m)	3.66 (2H, m)
2’	2.84 (2H, t, 7.5)	2.77 (2H, t, 7.1)
9-OH	5.91 (brs)	–
13-NH	8.15 (brs)	8.33 (1H, brs)
15-NH	9.63 (1H, t, 5.4)	9.56 (t, 5.4)

[a] overlapped with HOD

Table S7. ^{1}H NMR data for oroidin, keramadine, and 2-bromo-9,10-dihydrokeramadine in DMSO-d_6 (500 MHz).

position	**oroidin**	**keramadine**	**2-bromo-9,10-dihydrokeramadine**
	^{1}H (J in Hz)	^{1}H (J in Hz)	^{1}H (J in Hz)
1	12.74 (brs)	11.83 (brs)	12.67 (brs)
2	–	6.97 (brs)	–
4	6.98 (brs)	6.83 (brs)	6.91 (brs)
7	8.52 (t, 5.2)	8.46 (t, 5.7)	8.26 (brs)
8	3.94 (2H, t, 5.2)	4.00 (2H, t, 5.7)	3.25 (2H, m)
9	6.09 (dt, 16.2, 5.2)	5.83 (dt, 11.7, 5.7)	1.73 (2H, m)
10	6.20 (d, 16.2)	6.24 (d, 11.7)	2.49 (2H, m)
12	12.88 (brs)	–	–
14	12.20 (brs)	12.67 (brs)	12.33 (brs)
15	6.88 (brs)	7.09 (s)	6.69 (1H, brs)
N-Me		3.37 (3H, s)	3.32 (3H, s)
13-NH_2	7.68 (2H, brs)	7.80 (2H, brs)	7.64 (2H, brs)

Table S8. ^{1}H NMR data for nagelamide L in DMSO-d_6 (500 MHz).

position	^{1}H (J in Hz)	position	^{1}H (J in Hz)
1	12.70 (brs)	1'	12.67 (brs)
4	6.90 (s)	4'	6.94 (d, 2.2)
7	8.17 (2H, brs)	7'	8.44 (t, 5.7)
8	3.25 (2H, m)	8'	3.94 (2H, m)
9	5.53 (td, 8.8, 2.0)	9'	5.94 (dt, 15.8, 6.5)
10	4.54 (d, 8.8)	10'	6.42 (d, 15.8)
12	12.41 (brs)	12'	12.49 (brs)
14	13.09 (brs)	14'	12.53 (brs)
15	7.02 (brs)		
13-NH_2	7.64 (2H, brs)	13'-NH_2	7.75 (2H, brs)
